# Role and Clinical Application of Metagenomic Next-Generation Sequencing in Immunocompromised Patients With Acute Respiratory Failure During Veno-Venous Extracorporeal Membrane Oxygenation

**DOI:** 10.3389/fcimb.2022.877205

**Published:** 2022-08-12

**Authors:** Yang-Chao Zhao, Yan-Zhong Ding, Xi Zhao, Guo-Wei Fu, Ming-Jun Huang, Xing-Xing Li, Qian-Qian Sun, Ya-Bai Kan, Jun Li, Shi-Lei Wang, Wen-Tao Ma, Qin-Fu Xu, Qi-Long Liu, Hong-Bin Li

**Affiliations:** ^1^ Department of Extracorporeal Life Support Center, Department of Cardiac Surgery, The First Affiliated Hospital of Zhengzhou University, Zhengzhou, China; ^2^ Department of Cardiology, Cardiovascular Center, Henan Key Laboratory of Hereditary Cardiovascular Diseases, The First Affiliated Hospital of Zhengzhou University, Zhengzhou, China; ^3^ Department of Respiration, The First Affiliated Hospital of Zhengzhou University, Zhengzhou, China; ^4^ Department of Surgery ICU, The First Affiliated Hospital of Zhengzhou University, Zhengzhou, China

**Keywords:** metagenomic next-generation sequencing, routine test, extracorporeal membrane oxygenation, antibiotic treatment, immunocompromised patients

## Abstract

**Objectives:**

There are few studies of metagenomic next-generation sequencing (mNGS) in immunocompromised patients assisted by veno-venous extracorporeal membrane oxygenation (vv-ECMO). The present study is aimed to investigate the pathogen-detected effect and clinical therapy value of mNGS technologies in immunocompromised patients assisted by vv-ECMO.

**Methods:**

Our study retrospectively enrolled 46 immunocompromised patients supported by vv-ECMO from Jan 2017 to June 2021 at the First Affiliated Hospital of Zhengzhou University, respectively. Patients were divided into the deterioration group (Group D) (n = 31) and improvement group (Group I) (n = 15) according to their outcomes. Baseline characteristics and etiological data of patients during hospitalization of 2 groups were compared. The pathogens detected by mNGS and antibiotic regimens guided by mNGS in immunocompromised patients assisted by vv-ECMO were analyzed.

**Results:**

Compared with Group I, the deterioration patients showed a higher percentage of chronic obstructive pulmonary disease (COPD) (32.3% vs. 6.7%, *p* < 0.01) and were significantly older (47.77 ± 16.72 years vs. 32 ± 15.05 years, *p* < 0.01). Within 48 h of being ECMO assisted, the consistency of the samples detected by traditional culture and mNGS at the same time was good (traditional culture vs. mNGS detection, the positive rate of bronchoalveolar lavage fluid (BALF) culture: 26.1% vs. 30.4%; the positive rate of blood sample culture: 12.2% vs. 12.2%, *p* > 0.05). However, mNGS detected far more pathogen species and strains than conventional culture (30 strains vs. 78 strains, *p* < 0.01); the most popular pathogen was *Klebsiella pneumoniae*. Parts of patients had their antibiotic treatment adjustments, and the improvement patients showed less usage of broad-spectrum antibiotics.

**Conclusions:**

mNGS may play a relatively important role in detecting mixed pathogens and personalized antibiotic treatment in immunocompromised patients assisted by vv-ECMO.

## 1 Introduction

Immunosuppressive therapy is extensively used in patients with nephropathy and connective tissue disease, but severe side effects such as immunodeficiency, severe infection, and steroid-induced diabetes can occur (Doria et al., 2006; Rimes-Stigare et al., 2015). When immunodeficiency patients develop a severe pulmonary infection, the timely diagnosis of pathogenic microorganisms is difficult (Thong and Chan, 2019). Sepsis, hypoxia, and other factors can lead to multiple organ dysfunction and finally result in acute respiratory distress syndrome (ARDS) (McGregor et al., 2015).

Veno-venous extracorporeal membrane oxygenation (vv-ECMO) is increasingly used as a life-support technique in critically ill patients with reversible refractory respiratory failure and some other situations, such as organ transplantation (Professional Committee for Extracorporeal Life Support of Chinese Medical Doctor Association, 2018; Guglin et al., 2019). In recent years, with a deeper understanding of ECMO and the rapid improvement of ECMO accessories and operation methods, the survival rate of severely ill patients has also increased compared with before (Oh Tak et al., 2021; Kochanek et al., 2022). However, new issues and side effects have emerged. Immune suppression, long-term bed rest, physical weakness, and many invasive procedures, including continuous renal replacement therapy (CRRT) and tracheal intubation, could increase the rate of patients’ infection (Kühn et al., 2020). Infections are common complications in vv-ECMO patients and are associated with an increase in mortality (Burket et al., 1999).

Due to the high infection rate of vv-ECMO patients with low immunity (Dioverti et al., 2016; Nureki et al., 2020), the complex composition of pathogens, and the emergence of new, unknown, and drug-resistant pathogens, the difficulty of anti-infective treatment is increasing, resulting in huge pressure on doctors and patients. Timely and correct anti-infection treatment is the key to the successful treatment of vv-ECMO-supported immunocompromised patients with acute respiratory failure. In the case that the empirical treatment is ineffective, the causative pathogen is unknown, and the conventional microbial detection method has no positive indication, it is urgent for clinicians to find the causative pathogen quickly and accurately, give sensitive antibiotics for precise treatment, and improve the poor prognosis. At present, the commonly used pathogenic detection methods of pneumonia, such as morphological examination (smear and transmission electron microscopy), isolation and culture, and immunological methods, have their own advantages (Mazloomirad et al., 2021). However, for severe infection cases, the traditional methods have limitations in terms of detection time, positive rate, and specificity. Next-generation sequencing (NGS) technology, also known as high-throughput sequencing technology, is marked by the ability to sequence hundreds of thousands to millions of DNA molecules in parallel and generally shorter read lengths (Zhang et al., 2019). Metagenomic NGS (mNGS) is not based on culture, but the DNA of all microorganisms is directly extracted from clinical specimens (Yue et al., 2021). mNGS technology is widely used to explore the pathogens of difficult infectious diseases (Zhang et al., 2020).

Our present study reviewed 46 immunocompromised patients with acute respiratory failure who were treated with vv-ECMO in the First Affiliated Hospital of Zhengzhou University and analyzed the value of mNGS technology in the etiological diagnosis of patients and its guiding value for treatment plans.

## 2 Materials and Methods

### 2.1 Study Design and Population

**Figure 1:**
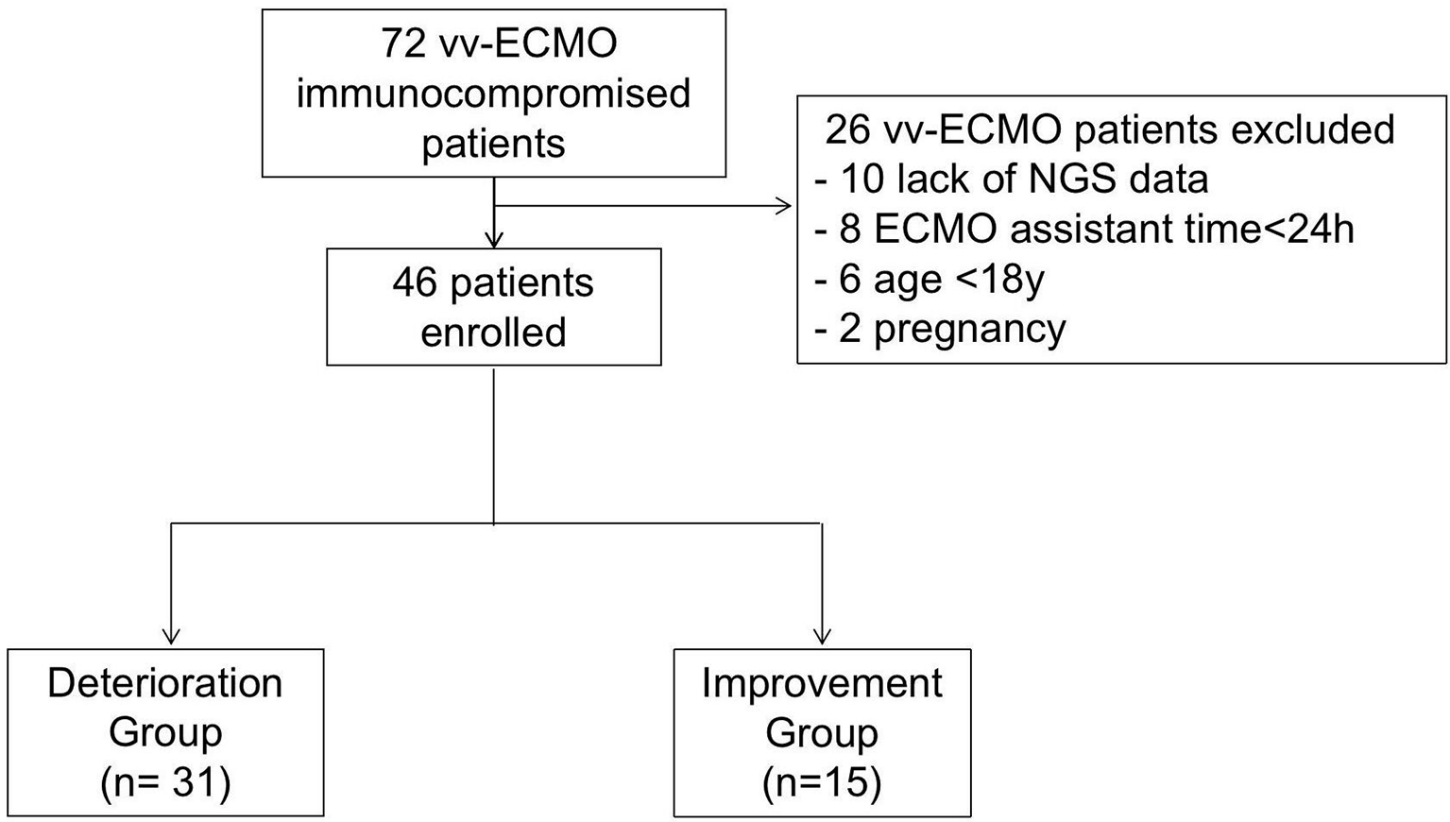
The flowchart.

The present study retrospectively enrolled immunocompromised patients supported by vv-ECMO from Jan 2017 to June 2021 at the First Affiliated Hospital of Zhengzhou University respectively. During this period, 72 immunocompromised patients had indications for vv-ECMO assistance. Among them, those who were excluded were 8 patients with ECMO assistant times of less than 24 h, 10 who lack NGS data, and patients younger than 18 years and pregnant women (**Figure 1**). Finally, 46 patients, including 27 men and 19 women, were enrolled and divided into the deterioration group (Group D) (n = 31) and improvement group (Group I) (n = 15) according to their discharge status at the weaning of vv-ECMO. Patients were considered immunocompromised if they have connective tissue disease, nephropathy, and other diseases that needed immunosuppressive therapy. The criteria for the improvement group were as follows: 1) successfully weaned from ECMO, 2) vital signs were stabilized and do not need mechanical ventilation and vasoactive drugs, and 3) the symptoms and signs of the primary disease disappeared or improved, and the chest CT or X-ray result improved. The criteria for the worsening group were as follows: 1) in-hospital death and 2) critical condition but chose to be discharged immediately. All enrolled patients’ whole blood samples and bronchoalveolar lavage fluid (BALF) were collected and routinely cultured before and within 48 h after being ECMO assisted and were sent for mNGS test within 48 h after being ECMO assisted independently. The baseline characteristics and clinical data were collected. The etiological data and antibiotic regimens in immunocompromised patients assisted by vv-ECMO were analyzed. The clinical data of the patients were obtained through the electronic medical record system.

### 2.2 Methods and Process of Next-Generation Sequencing

The whole blood samples or BALF was collected from the infected patient within 48 h of ECMO initiation, and then plasma was obtained by centrifugation at 4°C. About 300 μl of plasma was used to extract DNA by using TIANamp Micro DNA Kit following the manufacturer’s instructions. The extracted DNA was interrupted by enzyme digestion and enriched with magnetic beads for PCR amplification and end-repair. Agilent 2100 bioanalyzer (Agilent, Santa Clara, CA, USA) and ABI StepOnePlus Realtime PCR System were used for quality control of the DNA library; the qualified libraries were sequenced on the NextSeq 550Dx platform (Illumina, San Diego, CA, USA) using 75-bp sequencing read length.

Illumina Next 550 sequencer was used for the metagenomics sequence, and 15–20 samples that contain 1 negative control were loaded in each metagenomics sequencing batch. The internal reference, which is from *Arabidopsis thaliana*, was provided by sequencing manufacturers. The diagnosis report criteria contain the following: 1) internal reference should be detected; 2) to determine whether an organism was a contaminant from the laboratory, the ratio of read abundance in a sample to read abundance in the batch negative control and the mean read abundance of cumulative negative controls in the last month were used. Moreover, only if organisms were found >5-fold in samples than in controls, they would be kept.

Then the raw reads were removed from the low-quality reads to get clean reads, and the clean reads were aligned to the human genome to remove host sequence reads; after that, the reads were all mapped to the commercial pathogen database to be annotated and analyzed. The data that support the findings of this study have been deposited into CNGB Sequence Archive (CNSA) (Guo et al., 2020) of China National GeneBank DataBase (CNGBdb) (Chen et al., 2020) with accession number CNP0002988.

### 2.3 Extracorporeal Membrane Oxygenation Application

All patients were assisted by vv-ECMO, using the femoral vein-internal jugular vein, and the puncture method was percutaneous venous catheterization. ECMO equipment adopts ECMO system kit, including centrifugal pump, oxygenator, and its connecting pipeline, among which the centrifugal pump adopts Rotaflow pump head and controller from Medtronic and MAQUET; the oxygenator is divided into Medtronic and MAQUET adult oxygenator; the pipeline includes ECMO kit pipeline and ordinary PVC pipeline, and the cannulae are all arteriovenous thin-walled.

#### 2.3.1 Extracorporeal Membrane Oxygenation Management

1) Management of mechanical ventilation: to avoid or reduce the occurrence of ventilator-related lung injury to the greatest extent while promoting the recruitment of collapsed alveoli. 2) Anticoagulation: unfractionated heparin anticoagulation to maintain activated coagulation time (ACT) is 1.5 times the upper limit of normal, and the activated partial thromboplastin time (APTT) is 40–55 s. 3) Analgesia and sedation: adequate analgesia, sedation combined with muscle relaxants, combined with the patient’s human-machine synchronization, and the complete suppression of spontaneous breathing. 4) Flow management: adjust the flow according to the patient’s monitoring indicators to maintain blood oxygen saturation (SaO_2_) at 85%–95% and PaO_2_ above 60 mmHg; vv-ECMO pump flow to 2–3 L/min, close vv-ECMO airflow, under certain respiratory support (FiO_2_ < 50%, positive end-expiratory pressure ventilation (PEEP) ≤ 10 cmH_2_O, peak airway pressure<30 cmH_2_O), monitor for 2–4 h, if SaO_2_ > 95%, and PaCO_2_ < 50 mmHg; weaning of vv-ECMO can be considered.

### 2.4 Statistical Analysis

All collected data were statistically analyzed using SPSS 21.0 (IBM Corp., Armonk, NY, USA). Measurement data were expressed by mean ± SD, and the two groups were compared by ANOVA. Count data were expressed by frequency (composition ratio), and comparison between groups was performed by χ^2^ test or Fisher’s exact test. *p* < 0.05 indicates that the difference is statistically significant.

## 3 Results

### 3.1 Clinical and Biochemical Characteristics

The characteristics of enrolled patients are shown in **Table 1**, with 58.7% being male. Of the two groups, there was no significant difference in patients’ gender, body mass index (BMI), the system score before ECMO [Murray Lung Injury Score, sequential organ failure assessment (SOFA) score, CURB-65 Score, and APACHE II Score], and the baseline data of vital signs (heart rate, mean arterial pressure, and respiratory rate) (all *p* > 0.05, **Table 1**). Moreover, the PaO_2_/FiO_2_, PEEP and mechanical ventilation time before and after being ECMO assisted, the ECMO support time, the percentages of vasoactive drug used, pneumothorax, prone position ventilation, multiple organ dysfunction syndrome (MODS), and CRRT also showed no significant differences between the two groups (all *p* > 0.05, **Table 1**). However, the deterioration group was significantly older than the improvement group (47.77 ± 16.72 years vs. 32 ± 15.05 years, *p* < 0.01, **Table 1**), and the intensive care unit (ICU) stay time of the deterioration group was significantly shorter than that of the improvement group [13 (7–18) vs. 32 (20–38) days, *p* < 0.001, **Table 1**).

**Table 1:** Baseline characteristics of immunocompromised acute respiratory failure patients supported with vv-ECMO.

	Total(n = 46)	Group D(n = 31)	Group I(n = 15)
**Male gender**, n (%)	27 (58.7)	16 (51.4)	11 (73.3)
Age^**^ (year)	42.63 ± 17.68	47.77 ± 16.72	32 ± 15.05
**BMI** (kg/m^2^)	22.79 ± 3.03	23 ± 3.4	22.35 ± 2.08
**Comorbidities**, n (%)			
COPD^**^	11 (23.9)	10 (32.3)	1 (6.7)
Hypertension	6 (13)	4 (12.9)	2 (13.3)
Type 2 diabetes	3 (6.5)	3 (9.7)	0 (0)
Coronary heart disease	5 (10.9)	4 (12.9)	1 (6.7)
**Primary disease**, n (%)			
Connective tissue disease	30 (65.2)	21 (67.7)	9 (60)
IgA nephropathy	6 (13)	3 (9.7)	3 (20)
Other nephropathy types	10 (21.7)	7 (22.6)	3 (20)
**Murray Lung Injury Score**	3.14 ± 0.38	3.19 ± 0.36	3.03 ± 0.41
**SOFA Score**	12.02 ± 2.8	12.23 ± 3.11	11.6 ± 2.06
**CURB-65 Score**	2.02 ± 0.91	2.16 ± 0.90	1.73 ± 0.88
APACHE II **Score**	21.59 ± 7.77	22.23 ± 8.02	20.27 ± 7.32
**Heart rate**	111.41 ± 21.12	111.29 ± 21.67	111.67 ± 20.65
**Mean arterial pressure** (mmHg)	84.67 ± 17.23	86.13 ± 19.2	81.67 ± 12.22
**Respiratory rate**	25.41 ± 7.77	26.71 ± 8.28	22.73 ± 5.95
**PaO_2_/FiO_2_ before ECMO**	47 (41–55)	49 (41–54)	43 (38–60)
**PaO_2_/FiO_2_ after ECMO**	160 (132–210)	173 (140–219)	139 (127–191)
**PEEP before ECMO** (cmH_2_O)	10.54 ± 4.93	10.74 ± 5.27	10.13 ± 4.27
**PEEP after ECMO** (cmH_2_O)	9.15 ± 3.42	9.42 ± 3.71	8.6 ± 2.75
**Vasoactive drug**, n (%)	33 (71.7)	23 (74.2)	10 (66.7)
**Pneumothorax**, n (%)	9 (19.6)	5 (16.1)	4 (26.7)
**Prone position ventilation**, n (%)	11 (23.3)	8 (25.8)	3 (20)
**MODS**, n (%)	17 (37)	13 (41.9)	4 (26.7)
**CRRT**, n (%)	25 (54.3)	14 (45.2)	11 (73.3)
**Mechanical ventilation time before ECMO** (h)	31 (4–60)	43 (5–75)	21 (3–56)
**Mechanical ventilation time** (h)	222 (154–323)	212 (122–312)	266 (210–384)
**ECMO support time** (h)	300 (162–425)	264 (144–378)	432 (216–520)
ICU stay time^***^ (days)	17 (10–32)	13 (7–18)	32 (20–38)

Group D, deterioration group; Group I, improvement group; BMI, body mass index; COPD, chronic obstructive pulmonary disease; SOFA, sequential organ failure assessment score; PEEP, positive end-expiratory pressure; MODS, multiple organ dysfunction syndrome; CRRT, continuous renal replacement therapy; ECMO, extracorporeal membrane oxygenation; ICU, intensive care unit; vv-ECMO, veno-venous extracorporeal membrane oxygenation.

The data are shown as mean ± SD, median (interquartile 25–75), or n (percentage).

Significant difference (^**^ p < 0.01 and ^***^ p < 0.001).

As shown in **Table 1**, the percentage of chronic obstructive pulmonary disease (COPD) was significantly higher in the deterioration group (32.3%) than that in the improvement group (6.7%) (*p* < 0.01, **Table 1**). The primary diseases of vv-ECMO-supported patients were connective tissue disease (Group D vs. Group I: 67.7% vs. 60%), IgA nephropathy (9.7% vs. 20%), and other types of nephropathy (22.6% vs. 20%) (all *p* > 0.05, **Table 1**). The percentage of IgA nephropathy in the improvement group seemed higher than in the deterioration group, but there were no significant differences.

Moreover, compared with the improvement group, the deterioration group showed higher white blood cell (WBC) counts (14.05 ± 5.44 * 10^9^/L vs. 10.16 ± 4.78 * 10^9^/L, *p* < 0.05, **Table 2**) at the time during 24 h before being ECMO assisted, longer APTT [46.1 (36.8–62.6) s vs. 37.5 (31.1–47) s, *p* < 0.05, **Table 2**], and higher levels of total bilirubin (TB) [33.5 (15–101) μmol/L vs. 21.9 (17.7–33.9) μmol/L, *p* < 0.05, **Table 2**] and direct bilirubin (DB) [17.1 (6.7–75.5) μmol/L vs. 9.4 (2–11.8) μmol/L, *p* < 0.05, **Table 2**] at the time of ECMO weaning.

**Table 2:** Comparison of laboratory examinations at three different time points.

Variables	24 h before ECMO		48 h within ECMO		ECMO weaned	
	Group D	Group I	Group D	Group I	Group D	Group I
**WBC^#^** (*10^9^/L)	14.05 ± 5.44	10.16 ± 4.78	11.15 ± 5.68	10.25 ± 3.94	15.45 ± 12.11	12.16 ± 6.19
**Neutrophils** (*10^9^/L)	9.46 ± 6.17	8.67 ± 4.46	9.64 ± 5.65	9.20 ± 3.57	13.82 ± 11.32	10.60 ± 5.66
**ALT** (U/L)	42 (29–84)	69 (24–137)	43 (33–78)	50 (18–101)	44 (27–65)	31 (17–87)
**AST** (U/L)	73 (36–122)	91 (37–181)	68 (37–133)	65 (33–160)	56 (40–111)	51 (33–90)
**Albumin** (g/L)	29.99 ± 4.24	29.48 ± 4.97	28.65 ± 5.67	30.51 ± 4.62	35.51 ± 8.69	34.45 ± 4.36
**Globulin** (g/L)	28.49 ± 6.41	26.63 ± 7.15	27.25 ± 8.22	27.95 ± 6.93	31.29 ± 11.01	30.68 ± 11.57
**TB^&^** (μmol/L)	13.7 (8.8–23.8)	16.8 (13–25.1)	25 (10.8–43.4)	22.2 (15–33)	33.5 (15–101)	21.9 (17.7–33.9)
**DB^&^** (μmol/L)	6 (1.2–16.5)	9.2 (5–15)	10.1 (4–25.1)	10.3 (3.4–16.4)	17.1 (6.7–75.5)	9.4 (2–11.8)
**LDH** (U/L)	788 (387–1,299)	682 (408–1,112)	935 (443–1,764)	605 (381–840)	733 (432–1,500)	667 (388–980)
**NT-pro-BNP** (pg/ml)	680 (201–3,324)	1,908 (532–3,061)	1,872 (312–3,336)	1,603 (905.2–2,763)	867 (300–3,160)	630 (199.9–1,001)
**PT** (s)	12.34 ± 2.56	12.76 ± 2.69	13.49 ± 3.41	13.23 ± 2.54	15.56 ± 11.22	12.43 ± 1.63
**INR**	1.14 ± 0.30	1.23 ± 0.32	1.22 ± 0.31	1.17 ± 0.24	1.40 ± 1.03	1.17 ± 0.25
**APTT^&^** (s)	35 (29.5–40.5)	29.8 (27–33.2)	41.9 (36–81.7)	34.8 (29.8–122)	46.1 (36.8–62.6)	37.5 (31.1–47)
**Fibrinogen** (g/L)	4.09 ± 1.57	4.26 ± 1.58	3.56 ± 1.65	4.30 ± 1.63	3.49 ± 1.70	3.29 ± 1.21
**D-dimer** (mg/L)	3.32 (1.03–6.42)	2.7 (1.1–3.61)	2.42 (1–6.25)	3.27 (0.7–5.24)	2.97 (1.4–8.4)	7.52 (1–14.22)
**Procalcitonin** (ng/ml)	1.21 (0.4–3.32)	2.78 (0.58–4.89)	2.39 (1.1–14.4)	3.31 (1.8–32.6)	2.52 (0.61–17.7)	2.9 (0.48–3.14)
**CRP** (mg/L)	113 (67–200)	100 (70.11–266)	97 (43–187)	137.5 (61–206)	86 (56–196)	108 (63–132)
**ESR** (mm/h)	43.31 ± 25.47	37.37 ± 26.39	40.64 ± 31.03	45.32 ± 33.08	36.83 ± 32.76	33.42 ± 26.48
**PaO_2_** (mmHg)	50 (45–59)	63 (47–87.3)	80 (67–90)	79 (59–103)	69.8 (60–96.5)	88 (68–116)
**PaCO_2_** (mmHg)	37 (33.7–46)	36 (29.9–48)	38 (34–43)	38 (33–44)	39 (35.5–45)	40 (33.9–46)
**Lactate** (mmol/L)	1.6 (1.2–2.5)	2.1 (1.3–3)	2 (1.4–3.2)	2.1 (1.3–3.5)	2 (1.5–3.1)	1.8 (1.2–2.4)

Group D, deterioration group; Group I, improvement group; ECMO, extracorporeal membrane oxygenation; ALT, alanine aminotransferase; AST, aspartate aminotransferase; TB, total bilirubin; DB, direct bilirubin; LDH, lactic dehydrogenase; PT, prothrombin time; INR, international normalized ratio; APTT, activated partial thromboplastin time; CRP, C-reactive protein; ESR, erythrocyte sedimentation rate; WBC, white blood cells.

The data are shown as mean ± SD, median (interquartile 25–75), or n (percentage).

^#^Statistical significance in 24 h before ECMO.

^&^Statistical significance when ECMO was weaned.

### 3.2 Pathogens Detected by Metagenomic Next-Generation Sequencing Technology in Veno-Venous Extracorporeal Membrane Oxygenation-Assisted Immunocompromised Patients

**Figure 2:**
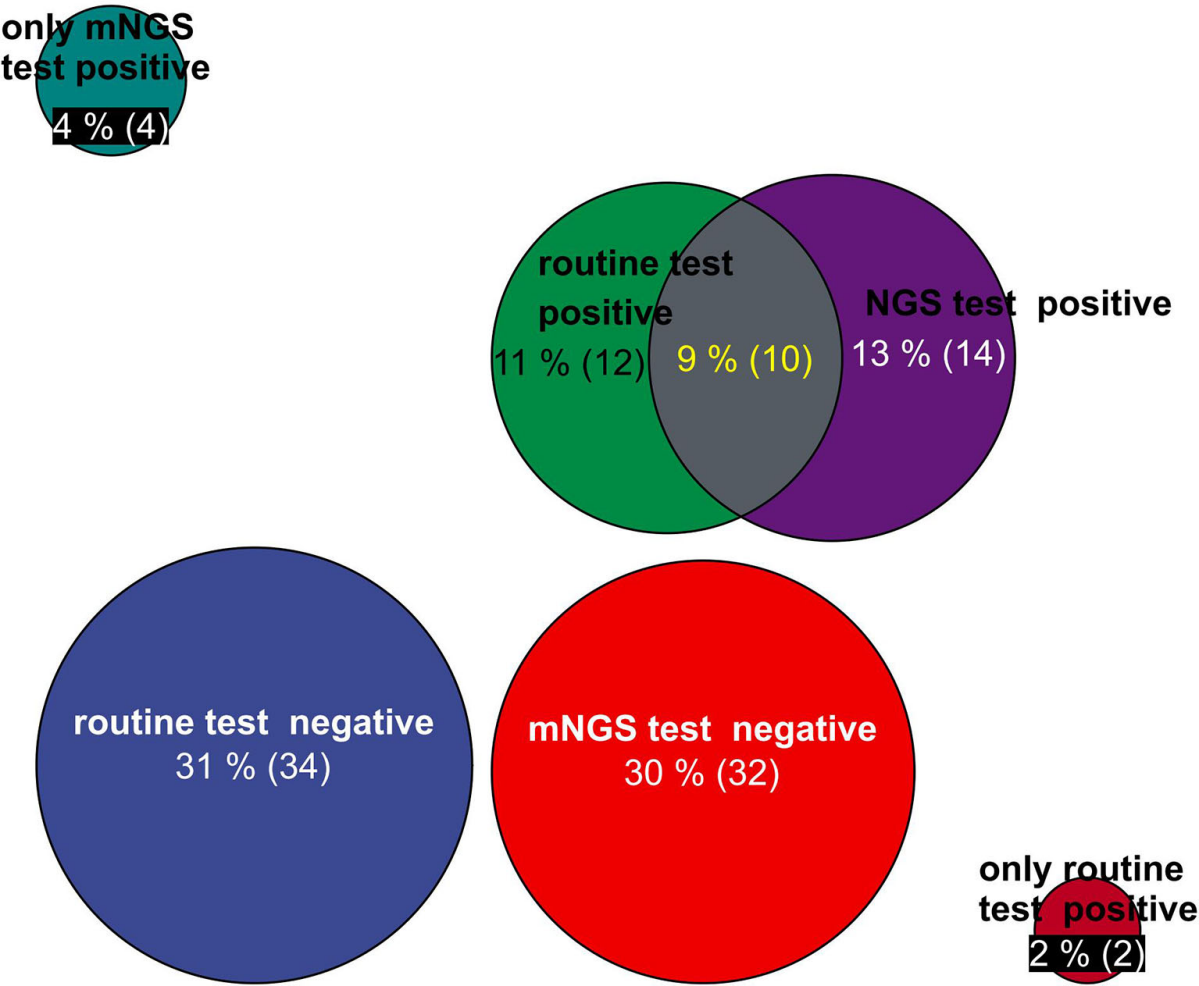
Circle chart of statistics on the number of patients detected by mNGS and conventional technology. The patients were divided into five groups for statistics: the number of positive patients only by routine test, the number of positive patients only by mNGS test, the number of positive patients by routine test and mNGS test, the number of negative patients by mNGS test, and the number of negative patients by routine test. mNGS, metagenomic next-generation sequencing.

The positivity rates of mNGS and culture test for both groups are illustrated in **Figure 2**. A total of 14 patients’ BALF and 5 patients’ blood samples tested positive for mNGS, of which 9 BALF samples and 3 blood samples were in the deterioration group and 5 BALF samples and 2 blood samples were in the improvement group (**Figure 2**).

In **Table 3**, the positive numbers of patients, the numbers of pathogens, and the numbers of different pathogen strains detected by mNGS and routine test from the BALF and blood samples of two groups are shown, but there was no significant difference between Group I and Group D.

**Table 3:** Pathogens detected by mNGS and routine test of immunocompromised patients supported with vv-ECMO.

	Group D(n = 31)			Group I(n = 15)		
	Positive patients	Pathogens	Strains	Positive patients	Pathogens	Strains
**24 h before ECMO** (routine test)						
BALF culture	5 (16.1)	6	10	3 (20.0)	3	3
Blood culture	2 (6.5)	3	3	1 (6.7)	1	1
**48 h within ECMO** (routine test)						
BALF culture	8 (25.8)	8	17	4 (26.7)	6	6
Blood culture	3 (9.7)	3	4	2 (13.3)	3	3
**48 h within ECMO** (mNGS test)						
BALF culture	9 (29.0)	27	47	5 (33.3)	17	21
Blood culture	3 (9.7)	6	6	2 (13.3)	3	3

mNGS, metagenomic next-generation sequencing; ECMO, extracorporeal membrane oxygenation; BALF, bronchoalveolar lavage fluid; vv-ECMO, veno-venous extracorporeal membrane oxygenation.

The data are shown as n (percentage).

**Figure 3:**
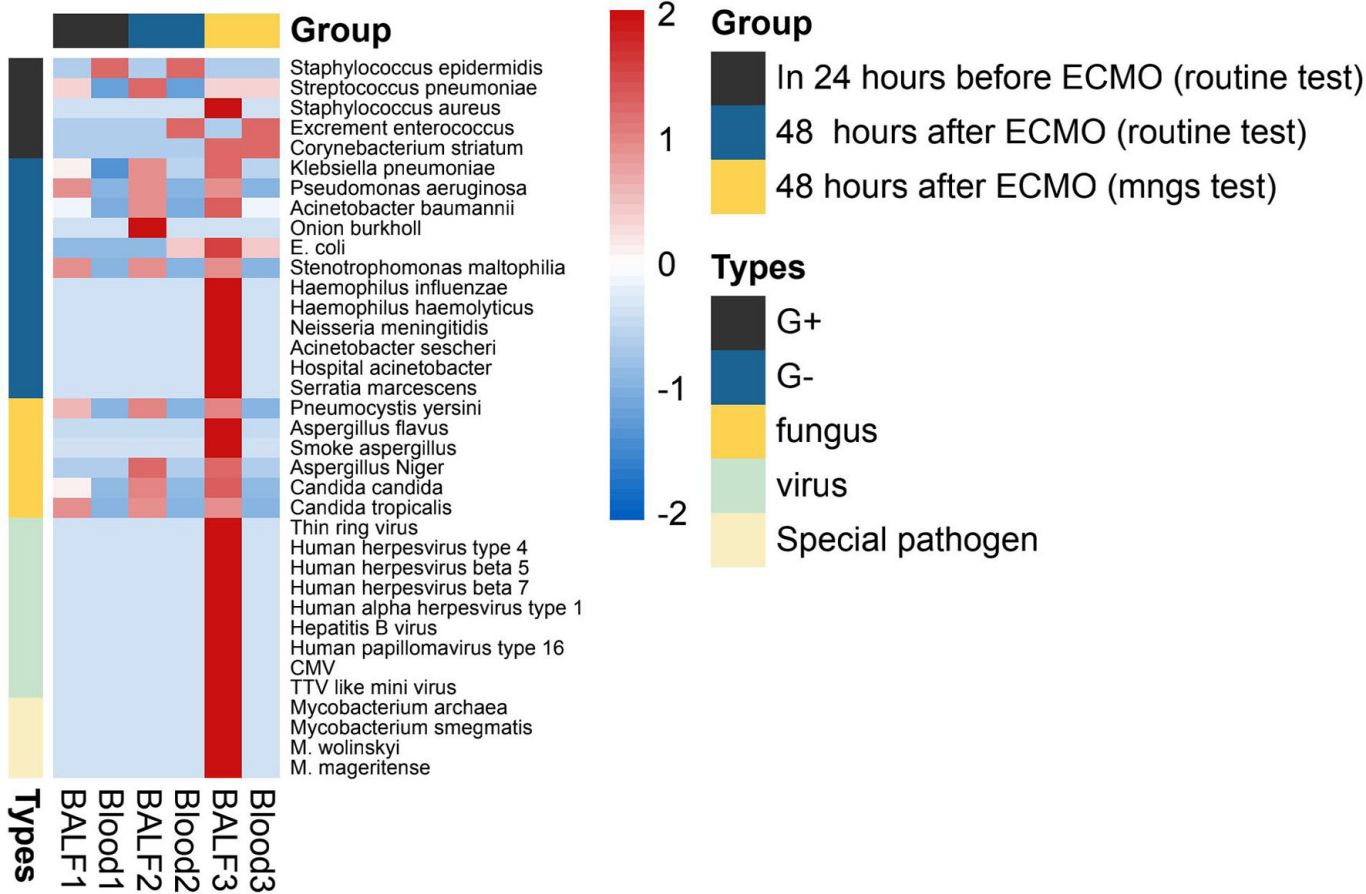
The heatmap of the pathogen species and strains detected by mNGS (within 48 h after ECMO) or routine test (24 h before and within 48 h after ECMO). The value of each pathogen represents the number of times detected in this group; after log10 conversion, the value is used for visual display. The pathogen was divided into five categories: G+ (Gram-positive bacteria), G− (Gram-negative bacteria), fungus, virus, and special pathogen. Patients of each group were tested for blood and BALF (bronchoalveolar lavage fluid) by mNGS or routine test. mNGS, metagenomic next-generation sequencing; ECMO, extracorporeal membrane oxygenation.

The details of the pathogen species and strains detected by mNGS (within 48 h after ECMO) or routine test (24 h before and within 48 h after ECMO) are shown in **Figure 3**. As shown in **Figure 3**, the spectrum of detected pathogens varied between the mNGS test and routine test of immunocompromised individuals supported by ECMO. The types of microorganisms detected by mNGS technology were significantly more plentiful than those of routine testing, and the detection efficiency of mNGS in viruses and special pathogens was better. The most common pathogens were *Klebsiella pneumoniae*, *Acinetobacter baumannii*, and Human betaherpesvirus 5.

### 3.3 Application of Metagenomic Next-Generation Sequencing in Immunocompromised Patients for Antibiotic Therapy

**Figure 4:**
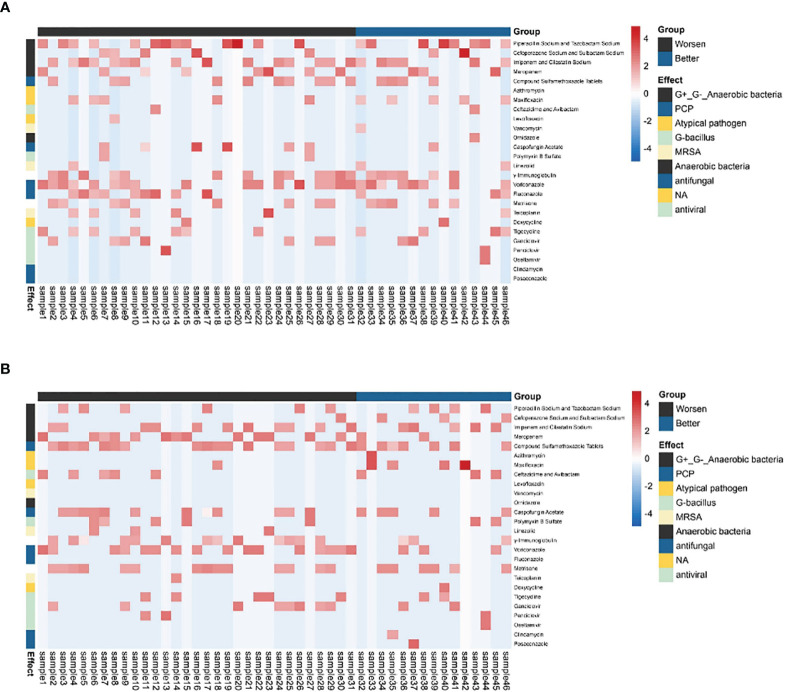
The antibiotic usage of patients. **(A)** The use of antibiotics before being assisted by the ECMO. **(B)** The use of antibiotics after ECMO was removed. The heatmaps were drawn using log10 (days of drug treatment), and all of the drugs were divided into eight categories according to their function. NA, no clear classification; ECMO, extracorporeal membrane oxygenation.

There was no significant difference in the prior antibiotic exposure prior to ECMO admission between both groups (**Table 4** and **Figure 4**). Moreover, there was no significant difference in medication between Group I and Group B (**Figure 4**). The antifungal treatment had changed significantly after mNGS test: the use of Caspofungin increased, while fluconazole was halted. Compared to the improvement patients, the deterioration patients used more voriconazole [16 (51.6) vs. 3 (20.0), *p* < 0.05, **Table 4**] and meropenem and imipenem [24 (77.4) vs. 7(46.7), *p* < 0.05, **Table 4**]. Moreover, the improvement patients showed less usage of broad-spectrum antibiotics (**Table 4**).

**Table 4:** Pathogens detected by mNGS and routine test of immunocompromised patients supported with vv-ECMO.

Antibiotics	Before mNGS test		After mNGS test	
	Group D	Group I	Group D	Group I
**Vancomycin**	0 (0)	1 (6.7)	0 (0)	0 (0)
**Linezolid**	5 (16.1)	1 (6.7)	1 (3.2)	0 (0)
**Teicoplanin**	1 (3.2)	2 (13.3)	3 (9.7)	0 (0)
**Tigecycline**	6 (19.4)	4 (26.7)	5 (16.1)	2 (13.3)
**Polymyxin B**	2 (6.5)	0 (0)	5 (16.1)	2 (13.3)
**Meropenem and Imipenem^&^**	22 (71.0)	9 (60.0)	24 (77.4)	7 (46.7)
**Ceftazidime/avibactam**	1 (3.2)	1 (6.7)	5 (16.1)	3 (20.0)
**Caspofungin**	6 (19.4)	1 (6.7)	12 (38.7)	5 (33.3)
**Voriconazole^&^**	17 (54.8)	8 (53.3)	16 (51.6)	3 (20.0)
**Fluconazole**	8 (25.8)	3 (20.0)	0 (0)	0 (0)

Group D, deterioration group; Group I, improvement group; mNGS, metagenomic next-generation sequencing; vv-ECMO, veno-venous extracorporeal membrane oxygenation.

The data are shown as n (percentage).

Statistical significance between Group D and Group I before mNGS test.

^&^Statistical significance between Group D and Group I after mNGS test.

## 4 Discussion

In the present study, we retrospectively analyzed the pathogen detection effect and clinical personalized antibiotic therapy value of mNGS technologies in a Chinese cohort of immunocompromised patients receiving vv-ECMO for acute respiratory failure.

Previous studies have found that infection is an important factor affecting the prognosis of ECMO patients (Steiner et al., 2001; Bif et al., 2017). The clinical diagnosis of infection in ECMO patients is a challenging topic because they always have signs of systemic inflammation, which may be triggered by the ECMO itself, and because the body temperature is controlled by the ECMO heat exchanger, fever is usually not obvious. Immunocompromised patients are often co-infected with multiple pathogens, and it is not easy to accurately detect mixed pathogens by routine clinical tests (Crotty et al., 2015). The immunocompromised patients assisted by vv-ECMO were often characterized as having high infection rates, mixed pathogen infection, poor prognosis, and even overuse of antibiotics. At present, the commonly used clinical methods to diagnose pathogens include G/GM experiments, bacterial culture, and quantitative PCR (qPCR) (Burket et al., 1999; Haneke et al., 2016). However, these methods usually have limitations. Conventional pathogen culture is currently the universal and necessary “gold standard” in clinical practice, but the culture time is long, and the positive rate is low. The test results of the G/GM experiment will be affected by many factors, its specificity and sensitivity are not high, and the incidence of false-positive and false-negative results is not low and can only be used for preliminary approximate discrimination of fungi. Another commonly used technique is qPCR, which is widely used but has low sensitivity. However, when the copy number of the virus is too low, it cannot be detected, early infection cannot be detected in time, and only specific pathogens can be detected. The common defect of these testing methods is that the number of specimens required for testing is large, and sufficient blood volume must be obtained. Therefore, there is a need for a clinical detection method that is different from the traditional one, which can detect pathogens early and accurately and avoid uncontrolled infections that exacerbate disease progression and seriously affect the prognosis of critically ill patients during ECMO.

NGS technology has the advantages of high detection sensitivity, short detection cycles, and a wide application range. This technology does not require the isolation and culture of pathogens, reduces the false-negative rate, and can analyze multiple pathogens at the same time, which greatly advances the treatment of specific pathogens. In summary, NGS technology not only solves many problems in traditional detection methods but also the pathogen detection rate of this technology is significantly better than that of traditional detection methods such as qPCR (Brown et al., 2018; Carpenter et al., 2019). A recent study found that in immunocompromised children, half of BALF samples previously detected as negative by routine test have potential pathogens detected by mNGS test (Zinter et al., 2019). Theoretically, NGS technology can detect the presence of pathogens, even all nucleotide sequences, which broadens the diagnostic scope of infections. That is, NGS technology has an absolute advantage in the diagnosis of mixed pathogen infection, especially in immunocompromised patients (Wang et al., 2019). Consistent with the above findings, our findings also showed that the mNGS test is more effective than a routine test and can detect multiple pathogenic microorganisms in mixed infections. In addition, *K. pneumoniae* and *A. baumannii* were the most popular pathogens detected in immunocompromised patients assisted by vv-ECMO in our present study. *K. pneumoniae* is a common opportunistic pathogen in ICUs. In the present study, *K. pneumoniae* was believed to be a pathogenic bacteria, for the following reasons: firstly, the microorganisms we detected are not common in the laboratory, and laboratory contamination bacteria are excluded; secondly, the sequenced microorganisms can cover different regions in the genome; finally, the microorganism is potentially pathogenic in a given clinical setting in a specific patient.

Immunocompromised patients assisted by vv-ECMO often have long hospital stays, heavy financial burdens, and high mortality rates. In addition to the difficult diagnosis of pathogens in immunocompromised patients with mixed infections, another problem is the frequent overuse of antibiotics, especially broad-spectrum antibiotics. Fortunately, mNGS may offer the probability for optimized antibiotic strategy and improved clinical excessive antibiotic use in immunocompromised patients assisted by vv-ECMO to alleviate antibiotic resistance and misuse of medical resources. In 2020, clinicians paid attention to the application value of mNGS in immunosuppressed patients with ARDS and found that mNGS detection can shorten the ICU stay time and the ventilation time and reduce the ICU cost significantly (Zhang et al., 2020). In our present study, mNGS has exhibited the effect of comprehensively evaluating and even effectively adjSusting the antimicrobial therapy in immunocompromised patients supported by vv-ECMO.

Our present study has some limitations. This study is a single-center retrospective study with a small sample size. Although we selected other more comprehensive measures such as the SOFA score, CURB-65 Score, and APACHE II Score to assess the severity of the disease in the two groups, these measures were not significantly different between the two groups; the older age of the Group D may affect patient outcomes. Then, most patients had experienced mechanical ventilation, which may have affected the pathogens’ results. Moreover, ECMO itself is an invasive operation, which may affect the treatment after being ECMO assisted.

## 5 Conclusion

In immunocompromised patients assisted by vv-ECMO, the pathogens detected by mNGS were consistent with the traditional culture (BALF and blood), but the pathogen species and strains detected by mNGS were richer. Some patients had changed their antibiotic treatment scheme. mNGS had an advantage in detecting mixed pathogens and personalized antibiotic treatment in immunocompromised patients assisted by vv-ECMO.

## Data Availability Statement

The data that support the findings of this study have been deposited into EMBL database with accession number is PRJEB55072.

## Ethics Statement

The studies involving human participants were reviewed and approved by the Ethics Committee of the First Affiliated Hospital of Zhengzhou University. The patients/participants provided their written informed consent to participate in this study. Written informed consent was obtained from the individual(s) for the publication of any potentially identifiable images or data included in this article.

## Author Contributions

Y-CZ and Y-ZD designed the study and wrote the first draft of the manuscript. XZ, G-WF, M-JH, S-LW, Y-BK, and JL verified data extraction and data analysis and reviewed the manuscript. W-TM, Q-FX, Q-LL, H-BL, X-XL, and Q-QS supervised the data acquisition, data analysis, and interpretation. All authors read and approved the final manuscript.

## Conflict of Interest

The authors declare that the research was conducted in the absence of any commercial or financial relationships that could be construed as a potential conflict of interest.

## Publisher’s Note

All claims expressed in this article are solely those of the authors and do not necessarily represent those of their affiliated organizations, or those of the publisher, the editors and the reviewers. Any product that may be evaluated in this article, or claim that may be made by its manufacturer, is not guaranteed or endorsed by the publisher.
