# Supplementary material for: Role and Clinical Application of Metagenomic Next-Generation Sequencing in Immunocompromised Patients With Acute Respiratory Failure During Veno-Venous Extracorporeal Membrane Oxygenation
Source: Front Cell Infect Microbiol. 2022 Aug 12;12:877205. doi: 10.3389/fcimb.2022.877205 (PMC9414489; doi:10.3389/fcimb.2022.877205)
Supplement: Supplementary file 2 [file DataSheet_2.pdf]

Supplyment 2 BRAF

SampleID	Group_D: mean rel. freq. (%)	Group_D: std. dev. (%)	Group_I: mean rel. freq. (%)	Group_I: std. dev. (%)	p-values	p-values (corrected)	Difference between means	95.0% lower CI	95.0% upper CI	Note
Acinetobacter_baumannii	2.06349206349	6.81061522315	2.97619047619	8.56398486306	0.723126181087	0.723126181087	-0.912698412698	-6.15868104185	4.33328421645	degenerate case: parent group had a count of zero
Acinetobacter_saimannii	0.952380952381	3.5634832255	0.892857142857	3.45802084483	0.957627403773	0.957627403773	0.0595238095238	-2.20663953801	2.32568715706	degenerate case: parent group had a count of zero
Aspergillus_flavus	0.555555555556	2.99175822619	3.125	12.1030729569	0.430119119984	0.430119119984	-2.56944444444	-9.29956738308	4.16067849419	degenerate case: parent group had a count of zero
Aspergillus_fumigatus	1.94444444444	7.35455683049	0.0	0.0	0.165185996458	0.165185996458	1.94444444444	-0.848739826264	4.73762871515	degenerate case: parent group had a count of zero
Aspergillus_niger	0.555555555556	2.99175822619	0.0	0.0	0.325581988016	0.325581988016	0.555555555556	-0.580683134518	1.69179424563	degenerate case: parent group had a count of zero
Candida_albicans	2.22222222222	7.11458248604	0.892857142857	3.45802084483	0.408974693851	0.408974693851	1.32936507937	-1.88455960566	4.54328976439	degenerate case: parent group had a count of zero
Candida_tropicalis	0.0	0.0	1.5625	6.05153647845	0.333170135915	0.333170135915	-1.5625	-4.89288991494	1.76788991494	degenerate case: parent group had a count of zero
CMV	0.0	0.0	1.25	4.84122918276	0.333170135915	0.333170135915	-1.25	-3.91431193195	1.41431193195	degenerate case: parent group had a count of zero
Corynebacterium_striatum	1.11111111111	5.98351645237	0.0	0.0	0.325581988016	0.325581988016	1.11111111111	-1.16136626904	3.38358849126	degenerate case: parent group had a count of zero
enterococcus_faecium	0.0	0.0	0.0	0.0	1.0	1.0	0.0	0.0	0.0	degenerate case: variance of both groups is zero
Escherichia_coli	0.952380952381	3.5634832255	0.892857142857	3.45802084483	0.957627403773	0.957627403773	0.0595238095238	-2.20663953801	2.32568715706	degenerate case: parent group had a count of zero
Haemophilus_haemolyticus	0.555555555556	2.99175822619	0.0	0.0	0.325581988016	0.325581988016	0.555555555556	-0.580683134518	1.69179424563	degenerate case: parent group had a count of zero
Haemophilus_influenzae	0.555555555556	2.99175822619	0.0	0.0	0.325581988016	0.325581988016	0.555555555556	-0.580683134518	1.69179424563	degenerate case: parent group had a count of zero
hepatitis_B_virus	2.05555555556	6.26103963284	0.0	0.0	0.087582102105	0.087582102105	2.05555555556	-0.322322245079	4.43343335619	degenerate case: parent group had a count of zero
Hospital _Acinetobacter	0.952380952381	3.5634832255	0.892857142857	3.45802084483	0.957627403773	0.957627403773	0.0595238095238	-2.20663953801	2.32568715706	degenerate case: parent group had a count of zero
human_beings_;Á_Herpesvirus1	0.0	0.0	1.25	4.84122918276	0.333170135915	0.333170135915	-1.25	-3.91431193195	1.41431193195	degenerate case: parent group had a count of zero
human_beings_;Á_Herpesvirus5	2.05555555556	6.26103963284	3.64583333333	9.7578093725	0.572493325633	0.572493325633	-1.59027777778	-7.35158762172	4.17103206616	degenerate case: parent group had a count of zero
human_beings_;Á_Herpesvirus7	0.0	0.0	1.25	4.84122918276	0.333170135915	0.333170135915	-1.25	-3.91431193195	1.41431193195	degenerate case: parent group had a count of zero
Human_herpesvirus4	0.47619047619	2.56436419387	0.0	0.0	0.325581988016	0.325581988016	0.47619047619	-0.497728401016	1.4501093534	degenerate case: parent group had a count of zero
Human_papillomavirus_type16	2.05555555556	6.26103963284	0.0	0.0	0.087582102105	0.087582102105	2.05555555556	-0.322322245079	4.43343335619	degenerate case: parent group had a count of zero
klebsiella_pneumoniae	2.61904761905	7.28301641096	2.14285714286	5.7587555345	0.814029401053	0.814029401053	0.47619047619	-3.59640727362	4.548788226	degenerate case: parent group had a count of zero
M.mageritense	0.555555555556	2.99175822619	0.0	0.0	0.325581988016	0.325581988016	0.555555555556	-0.580683134518	1.69179424563	degenerate case: parent group had a count of zero
M.wolinskyi	0.555555555556	2.99175822619	0.0	0.0	0.325581988016	0.325581988016	0.555555555556	-0.580683134518	1.69179424563	degenerate case: parent group had a count of zero
Microcyclic_virus	1.5873015873	6.42808150201	4.6875	13.1658590206	0.400379868091	0.400379868091	-3.1001984127	-10.6471385569	4.4467417315	degenerate case: parent group had a count of zero
Mycobacterium_Archaea	0.555555555556	2.99175822619	0.0	0.0	0.325581988016	0.325581988016	0.555555555556	-0.580683134518	1.69179424563	degenerate case: parent group had a count of zero
Mycobacterium_smegmatis	0.555555555556	2.99175822619	0.0	0.0	0.325581988016	0.325581988016	0.555555555556	-0.580683134518	1.69179424563	degenerate case: parent group had a count of zero
Neisseria_meningitidis	0.555555555556	2.99175822619	0.0	0.0	0.325581988016	0.325581988016	0.555555555556	-0.580683134518	1.69179424563	degenerate case: parent group had a count of zero
Pneumocystis_jiroveci	1.22222222222	4.59333924635	2.08333333333	8.0687153046	0.706082851007	0.706082851007	-0.861111111111	-5.55464686584	3.83242464361	degenerate case: parent group had a count of zero
Pseudomonas_aeruginosa	0.555555555556	2.99175822619	0.0	0.0	0.325581988016	0.325581988016	0.555555555556	-0.580683134518	1.69179424563	degenerate case: parent group had a count of zero
Serratia_marcescens	0.952380952381	3.5634832255	0.892857142857	3.45802084483	0.957627403773	0.957627403773	0.0595238095238	-2.20663953801	2.32568715706	degenerate case: parent group had a count of zero
Staphylococcus_aureus	0.666666666667	3.59010987142	0.0	0.0	0.325581988016	0.325581988016	0.666666666667	-0.696819761422	2.03015309476	degenerate case: parent group had a count of zero
Stenotrophomonas_maltophilia	0.0	0.0	1.25	4.84122918276	0.333170135915	0.333170135915	-1.25	-3.91431193195	1.41431193195	degenerate case: parent group had a count of zero
Streptococcus_pneumoniae	0.555555555556	2.99175822619	0.0	0.0	0.325581988016	0.325581988016	0.555555555556	-0.580683134518	1.69179424563	degenerate case: parent group had a count of zero
TTVlikeminivirus	0.0	0.0	1.5625	6.05153647845	0.333170135915	0.333170135915	-1.5625	-4.89288991494	1.76788991494	degenerate case: parent group had a count of zero

SampleID	Patient1	Patient1: parent seq. count	Patient1: rel. freq. (%)	Patient10	Patient10: parent seq. count	Patient10: rel. freq. (%)	Patient11	Patient11: parent seq. count	Patient11: rel. freq. (%)	Patient12	Patient12: parent seq. count	Patient12: rel. freq. (%)	Patient13	Patient13: parent seq. count	Patient13: rel. freq. (%)	Patient14	Patient14: parent seq. count	Patient14: rel. freq. (%)
Acinetobacter_baumannii	0.0	0.0	0.0	0.0	0.0	0.0	0.0	0.0	0.0	0.0	1.0	0.0	0.0	0.0	0.0	0.0	1.0	0.0
Acinetobacter_saimannii	0.0	0.0	0.0	0.0	0.0	0.0	0.0	0.0	0.0	0.0	1.0	0.0	0.0	0.0	0.0	0.0	1.0	0.0
Aspergillus_flavus	0.0	0.0	0.0	0.0	0.0	0.0	0.0	0.0	0.0	0.0	1.0	0.0	0.0	0.0	0.0	0.0	1.0	0.0
Aspergillus_fumigatus	0.0	0.0	0.0	0.0	0.0	0.0	0.0	0.0	0.0	0.0	1.0	0.0	0.0	0.0	0.0	0.25	1.0	25.0
Aspergillus_niger	0.0	0.0	0.0	0.0	0.0	0.0	0.0	0.0	0.0	0.0	1.0	0.0	0.0	0.0	0.0	0.0	1.0	0.0
Candida_albicans	0.0	0.0	0.0	0.0	0.0	0.0	0.0	0.0	0.0	0.0	1.0	0.0	0.0	0.0	0.0	0.0	1.0	0.0
Candida_tropicalis	0.0	0.0	0.0	0.0	0.0	0.0	0.0	0.0	0.0	0.0	1.0	0.0	0.0	0.0	0.0	0.0	1.0	0.0
CMV	0.0	0.0	0.0	0.0	0.0	0.0	0.0	0.0	0.0	0.0	1.0	0.0	0.0	0.0	0.0	0.0	1.0	0.0
Corynebacterium_striatum	0.0	0.0	0.0	0.0	0.0	0.0	0.0	0.0	0.0	0.0	1.0	0.0	0.0	0.0	0.0	0.0	1.0	0.0
enterococcus_faecium	0.0	0.0	0.0	0.0	0.0	0.0	0.0	0.0	0.0	0.0	1.0	0.0	0.0	0.0	0.0	0.0	1.0	0.0
Escherichia_coli	0.0	0.0	0.0	0.0	0.0	0.0	0.0	0.0	0.0	0.0	1.0	0.0	0.0	0.0	0.0	0.0	1.0	0.0
Haemophilus_haemolyticus	0.0	0.0	0.0	0.0	0.0	0.0	0.0	0.0	0.0	0.0	1.0	0.0	0.0	0.0	0.0	0.0	1.0	0.0
Haemophilus_influenzae	0.0	0.0	0.0	0.0	0.0	0.0	0.0	0.0	0.0	0.0	1.0	0.0	0.0	0.0	0.0	0.0	1.0	0.0
hepatitis_B_virus	0.0	0.0	0.0	0.0	0.0	0.0	0.0	0.0	0.0	0.2	1.0	20.0	0.0	0.0	0.0	0.25	1.0	25.0
Hospital _Acinetobacter	0.0	0.0	0.0	0.0	0.0	0.0	0.0	0.0	0.0	0.0	1.0	0.0	0.0	0.0	0.0	0.0	1.0	0.0
human_beings_;Á_Herpesvirus1	0.0	0.0	0.0	0.0	0.0	0.0	0.0	0.0	0.0	0.0	1.0	0.0	0.0	0.0	0.0	0.0	1.0	0.0
human_beings_;Á_Herpesvirus5	0.0	0.0	0.0	0.0	0.0	0.0	0.0	0.0	0.0	0.2	1.0	20.0	0.0	0.0	0.0	0.25	1.0	25.0
human_beings_;Á_Herpesvirus7	0.0	0.0	0.0	0.0	0.0	0.0	0.0	0.0	0.0	0.0	1.0	0.0	0.0	0.0	0.0	0.0	1.0	0.0
Human_herpesvirus4	0.0	0.0	0.0	0.0	0.0	0.0	0.0	0.0	0.0	0.0	1.0	0.0	0.0	0.0	0.0	0.0	1.0	0.0
Human_papillomavirus_type16	0.0	0.0	0.0	0.0	0.0	0.0	0.0	0.0	0.0	0.2	1.0	20.0	0.0	0.0	0.0	0.25	1.0	25.0
klebsiella_pneumoniae	0.0	0.0	0.0	0.0	0.0	0.0	0.0	0.0	0.0	0.0	1.0	0.0	0.0	0.0	0.0	0.0	1.0	0.0
M.mageritense	0.0	0.0	0.0	0.0	0.0	0.0	0.0	0.0	0.0	0.0	1.0	0.0	0.0	0.0	0.0	0.0	1.0	0.0
M.wolinskyi	0.0	0.0	0.0	0.0	0.0	0.0	0.0	0.0	0.0	0.0	1.0	0.0	0.0	0.0	0.0	0.0	1.0	0.0
Microcyclic_virus	0.0	0.0	0.0	0.0	0.0	0.0	0.0	0.0	0.0	0.0	1.0	0.0	0.0	0.0	0.0	0.0	1.0	0.0
Mycobacterium_Archaea	0.0	0.0	0.0	0.0	0.0	0.0	0.0	0.0	0.0	0.0	1.0	0.0	0.0	0.0	0.0	0.0	1.0	0.0
Mycobacterium_smegmatis	0.0	0.0	0.0	0.0	0.0	0.0	0.0	0.0	0.0	0.0	1.0	0.0	0.0	0.0	0.0	0.0	1.0	0.0
Neisseria_meningitidis	0.0	0.0	0.0	0.0	0.0	0.0	0.0	0.0	0.0	0.0	1.0	0.0	0.0	0.0	0.0	0.0	1.0	0.0
Pneumocystis_jiroveci	0.0	0.0	0.0	0.0	0.0	0.0	0.0	0.0	0.0	0.2	1.0	20.0	0.0	0.0	0.0	0.0	1.0	0.0
Pseudomonas_aeruginosa	0.0	0.0	0.0	0.0	0.0	0.0	0.0	0.0	0.0	0.0	1.0	0.0	0.0	0.0	0.0	0.0	1.0	0.0
Serratia_marcescens	0.0	0.0	0.0	0.0	0.0	0.0	0.0	0.0	0.0	0.0	1.0	0.0	0.0	0.0	0.0	0.0	1.0	0.0
Staphylococcus_aureus	0.0	0.0	0.0	0.0	0.0	0.0	0.0	0.0	0.0	0.2	1.0	20.0	0.0	0.0	0.0	0.0	1.0	0.0
Stenotrophomonas_maltophilia	0.0	0.0	0.0	0.0	0.0	0.0	0.0	0.0	0.0	0.0	1.0	0.0	0.0	0.0	0.0	0.0	1.0	0.0
Streptococcus_pneumoniae	0.0	0.0	0.0	0.0	0.0	0.0	0.0	0.0	0.0	0.0	1.0	0.0	0.0	0.0	0.0	0.0	1.0	0.0
TTVlikeminivirus	0.0	0.0	0.0	0.0	0.0	0.0	0.0	0.0	0.0	0.0	1.0	0.0	0.0	0.0	0.0	0.0	1.0	0.0

SampleID	Patient15	Patient15: parent seq. count	Patient15: rel. freq. (%)	Patient16	Patient16: parent seq. count	Patient16: rel. freq. (%)	Patient17	Patient17: parent seq. count	Patient17: rel. freq. (%)	Patient18	Patient18: parent seq. count	Patient18: rel. freq. (%)	Patient19	Patient19: parent seq. count	Patient19: rel. freq. (%)	Patient2	Patient2: parent seq. count	Patient2: rel. freq. (%)
Acinetobacter_baumannii	0.0	0.0	0.0	0.143	1.001	14.2857142857	0.0	0.0	0.0	0.0	0.0	0.0	0.0	0.0	0.0	0.0	0.0	0.0
Acinetobacter_saimannii	0.0	0.0	0.0	0.143	1.001	14.2857142857	0.0	0.0	0.0	0.0	0.0	0.0	0.0	0.0	0.0	0.0	0.0	0.0
Aspergillus_flavus	0.0	0.0	0.0	0.0	1.001	0.0	0.0	0.0	0.0	0.0	0.0	0.0	0.0	0.0	0.0	0.0	0.0	0.0
Aspergillus_fumigatus	0.0	0.0	0.0	0.0	1.001	0.0	0.0	0.0	0.0	0.0	0.0	0.0	0.0	0.0	0.0	0.0	0.0	0.0
Aspergillus_niger	0.0	0.0	0.0	0.0	1.001	0.0	0.0	0.0	0.0	0.0	0.0	0.0	0.0	0.0	0.0	0.0	0.0	0.0
Candida_albicans	0.0	0.0	0.0	0.0	1.001	0.0	0.0	0.0	0.0	0.0	0.0	0.0	0.0	0.0	0.0	0.0	0.0	0.0
Candida_tropicalis	0.0	0.0	0.0	0.0	1.001	0.0	0.0	0.0	0.0	0.0	0.0	0.0	0.0	0.0	0.0	0.0	0.0	0.0
CMV	0.0	0.0	0.0	0.0	1.001	0.0	0.0	0.0	0.0	0.0	0.0	0.0	0.0	0.0	0.0	0.0	0.0	0.0
Corynebacterium_striatum	0.0	0.0	0.0	0.0	1.001	0.0	0.0	0.0	0.0	0.0	0.0	0.0	0.0	0.0	0.0	0.0	0.0	0.0
enterococcus_faecium	0.0	0.0	0.0	0.0	1.001	0.0	0.0	0.0	0.0	0.0	0.0	0.0	0.0	0.0	0.0	0.0	0.0	0.0
Escherichia_coli	0.0	0.0	0.0	0.143	1.001	14.2857142857	0.0	0.0	0.0	0.0	0.0	0.0	0.0	0.0	0.0	0.0	0.0	0.0
Haemophilus_haemolyticus	0.0	0.0	0.0	0.0	1.001	0.0	0.0	0.0	0.0	0.0	0.0	0.0	0.0	0.0	0.0	0.0	0.0	0.0
Haemophilus_influenzae	0.0	0.0	0.0	0.0	1.001	0.0	0.0	0.0	0.0	0.0	0.0	0.0	0.0	0.0	0.0	0.0	0.0	0.0
hepatitis_B_virus	0.0	0.0	0.0	0.0	1.001	0.0	0.0	0.0	0.0	0.0	0.0	0.0	0.0	0.0	0.0	0.0	0.0	0.0
Hospital _Acinetobacter	0.0	0.0	0.0	0.143	1.001	14.2857142857	0.0	0.0	0.0	0.0	0.0	0.0	0.0	0.0	0.0	0.0	0.0	0.0
human_beings_;Á_Herpesvirus1	0.0	0.0	0.0	0.0	1.001	0.0	0.0	0.0	0.0	0.0	0.0	0.0	0.0	0.0	0.0	0.0	0.0	0.0
human_beings_;Á_Herpesvirus5	0.0	0.0	0.0	0.0	1.001	0.0	0.0	0.0	0.0	0.0	0.0	0.0	0.0	0.0	0.0	0.0	0.0	0.0
human_beings_;Á_Herpesvirus7	0.0	0.0	0.0	0.0	1.001	0.0	0.0	0.0	0.0	0.0	0.0	0.0	0.0	0.0	0.0	0.0	0.0	0.0
Human_herpesvirus4	0.0	0.0	0.0	0.0	1.001	0.0	0.0	0.0	0.0	0.0	0.0	0.0	0.0	0.0	0.0	0.0	0.0	0.0
Human_papillomavirus_type16	0.0	0.0	0.0	0.0	1.001	0.0	0.0	0.0	0.0	0.0	0.0	0.0	0.0	0.0	0.0	0.0	0.0	0.0
klebsiella_pneumoniae	0.0	0.0	0.0	0.143	1.001	14.2857142857	0.0	0.0	0.0	0.0	0.0	0.0	0.0	0.0	0.0	0.0	0.0	0.0
M.mageritense	0.0	0.0	0.0	0.0	1.001	0.0	0.0	0.0	0.0	0.0	0.0	0.0	0.0	0.0	0.0	0.0	0.0	0.0
M.wolinskyi	0.0	0.0	0.0	0.0	1.001	0.0	0.0	0.0	0.0	0.0	0.0	0.0	0.0	0.0	0.0	0.0	0.0	0.0
Microcyclic_virus	0.0	0.0	0.0	0.143	1.001	14.2857142857	0.0	0.0	0.0	0.0	0.0	0.0	0.0	0.0	0.0	0.0	0.0	0.0
Mycobacterium_Archaea	0.0	0.0	0.0	0.0	1.001	0.0	0.0	0.0	0.0	0.0	0.0	0.0	0.0	0.0	0.0	0.0	0.0	0.0
Mycobacterium_smegmatis	0.0	0.0	0.0	0.0	1.001	0.0	0.0	0.0	0.0	0.0	0.0	0.0	0.0	0.0	0.0	0.0	0.0	0.0
Neisseria_meningitidis	0.0	0.0	0.0	0.0	1.001	0.0	0.0	0.0	0.0	0.0	0.0	0.0	0.0	0.0	0.0	0.0	0.0	0.0
Pneumocystis_jiroveci	0.0	0.0	0.0	0.0	1.001	0.0	0.0	0.0	0.0	0.0	0.0	0.0	0.0	0.0	0.0	0.0	0.0	0.0
Pseudomonas_aeruginosa	0.0	0.0	0.0	0.0	1.001	0.0	0.0	0.0	0.0	0.0	0.0	0.0	0.0	0.0	0.0	0.0	0.0	0.0
Serratia_marcescens	0.0	0.0	0.0	0.143	1.001	14.2857142857	0.0	0.0	0.0	0.0	0.0	0.0	0.0	0.0	0.0	0.0	0.0	0.0
Staphylococcus_aureus	0.0	0.0	0.0	0.0	1.001	0.0	0.0	0.0	0.0	0.0	0.0	0.0	0.0	0.0	0.0	0.0	0.0	0.0
Stenotrophomonas_maltophilia	0.0	0.0	0.0	0.0	1.001	0.0	0.0	0.0	0.0	0.0	0.0	0.0	0.0	0.0	0.0	0.0	0.0	0.0
Streptococcus_pneumoniae	0.0	0.0	0.0	0.0	1.001	0.0	0.0	0.0	0.0	0.0	0.0	0.0	0.0	0.0	0.0	0.0	0.0	0.0
TTVlikeminivirus	0.0	0.0	0.0	0.0	1.001	0.0	0.0	0.0	0.0	0.0	0.0	0.0	0.0	0.0	0.0	0.0	0.0	0.0

SampleID	Patient20	Patient20: parent seq. count	Patient20: rel. freq. (%)	Patient21	Patient21: parent seq. count	Patient21: rel. freq. (%)	Patient22	Patient22: parent seq. count	Patient22: rel. freq. (%)	Patient23	Patient23: parent seq. count	Patient23: rel. freq. (%)	Patient24	Patient24: parent seq. count	Patient24: rel. freq. (%)	Patient25	Patient25: parent seq. count	Patient25: rel. freq. (%)
Acinetobacter_baumannii	0.143	1.001	14.2857142857	0.0	0.0	0.0	0.0	0.0	0.0	0.0	1.002	0.0	0.0	0.0	0.0	0.0	0.0	0.0
Acinetobacter_saimannii	0.143	1.001	14.2857142857	0.0	0.0	0.0	0.0	0.0	0.0	0.0	1.002	0.0	0.0	0.0	0.0	0.0	0.0	0.0
Aspergillus_flavus	0.0	1.001	0.0	0.0	0.0	0.0	0.0	0.0	0.0	0.0	1.002	0.0	0.0	0.0	0.0	0.0	0.0	0.0
Aspergillus_fumigatus	0.0	1.001	0.0	0.0	0.0	0.0	0.0	0.0	0.0	0.0	1.002	0.0	0.0	0.0	0.0	0.0	0.0	0.0
Aspergillus_niger	0.0	1.001	0.0	0.0	0.0	0.0	0.0	0.0	0.0	0.0	1.002	0.0	0.0	0.0	0.0	0.0	0.0	0.0
Candida_albicans	0.0	1.001	0.0	0.0	0.0	0.0	0.0	0.0	0.0	0.167	1.002	16.6666666667	0.0	0.0	0.0	0.0	0.0	0.0
Candida_tropicalis	0.0	1.001	0.0	0.0	0.0	0.0	0.0	0.0	0.0	0.0	1.002	0.0	0.0	0.0	0.0	0.0	0.0	0.0
CMV	0.0	1.001	0.0	0.0	0.0	0.0	0.0	0.0	0.0	0.0	1.002	0.0	0.0	0.0	0.0	0.0	0.0	0.0
Corynebacterium_striatum	0.0	1.001	0.0	0.0	0.0	0.0	0.0	0.0	0.0	0.0	1.002	0.0	0.0	0.0	0.0	0.0	0.0	0.0
enterococcus_faecium	0.0	1.001	0.0	0.0	0.0	0.0	0.0	0.0	0.0	0.0	1.002	0.0	0.0	0.0	0.0	0.0	0.0	0.0
Escherichia_coli	0.143	1.001	14.2857142857	0.0	0.0	0.0	0.0	0.0	0.0	0.0	1.002	0.0	0.0	0.0	0.0	0.0	0.0	0.0
Haemophilus_haemolyticus	0.0	1.001	0.0	0.0	0.0	0.0	0.0	0.0	0.0	0.0	1.002	0.0	0.0	0.0	0.0	0.0	0.0	0.0
Haemophilus_influenzae	0.0	1.001	0.0	0.0	0.0	0.0	0.0	0.0	0.0	0.0	1.002	0.0	0.0	0.0	0.0	0.0	0.0	0.0
hepatitis_B_virus	0.0	1.001	0.0	0.0	0.0	0.0	0.0	0.0	0.0	0.0	1.002	0.0	0.0	0.0	0.0	0.0	0.0	0.0
Hospital _Acinetobacter	0.143	1.001	14.2857142857	0.0	0.0	0.0	0.0	0.0	0.0	0.0	1.002	0.0	0.0	0.0	0.0	0.0	0.0	0.0
human_beings_;Á_Herpesvirus1	0.0	1.001	0.0	0.0	0.0	0.0	0.0	0.0	0.0	0.0	1.002	0.0	0.0	0.0	0.0	0.0	0.0	0.0
human_beings_;Á_Herpesvirus5	0.0	1.001	0.0	0.0	0.0	0.0	0.0	0.0	0.0	0.0	1.002	0.0	0.0	0.0	0.0	0.0	0.0	0.0
human_beings_;Á_Herpesvirus7	0.0	1.001	0.0	0.0	0.0	0.0	0.0	0.0	0.0	0.0	1.002	0.0	0.0	0.0	0.0	0.0	0.0	0.0
Human_herpesvirus4	0.143	1.001	14.2857142857	0.0	0.0	0.0	0.0	0.0	0.0	0.0	1.002	0.0	0.0	0.0	0.0	0.0	0.0	0.0
Human_papillomavirus_type16	0.0	1.001	0.0	0.0	0.0	0.0	0.0	0.0	0.0	0.0	1.002	0.0	0.0	0.0	0.0	0.0	0.0	0.0
klebsiella_pneumoniae	0.143	1.001	14.2857142857	0.0	0.0	0.0	0.0	0.0	0.0	0.0	1.002	0.0	0.0	0.0	0.0	0.0	0.0	0.0
M.mageritense	0.0	1.001	0.0	0.0	0.0	0.0	0.0	0.0	0.0	0.167	1.002	16.6666666667	0.0	0.0	0.0	0.0	0.0	0.0
M.wolinskyi	0.0	1.001	0.0	0.0	0.0	0.0	0.0	0.0	0.0	0.167	1.002	16.6666666667	0.0	0.0	0.0	0.0	0.0	0.0
Microcyclic_virus	0.0	1.001	0.0	0.0	0.0	0.0	0.0	0.0	0.0	0.0	1.002	0.0	0.0	0.0	0.0	0.0	0.0	0.0
Mycobacterium_Archaea	0.0	1.001	0.0	0.0	0.0	0.0	0.0	0.0	0.0	0.167	1.002	16.6666666667	0.0	0.0	0.0	0.0	0.0	0.0
Mycobacterium_smegmatis	0.0	1.001	0.0	0.0	0.0	0.0	0.0	0.0	0.0	0.167	1.002	16.6666666667	0.0	0.0	0.0	0.0	0.0	0.0
Neisseria_meningitidis	0.0	1.001	0.0	0.0	0.0	0.0	0.0	0.0	0.0	0.0	1.002	0.0	0.0	0.0	0.0	0.0	0.0	0.0
Pneumocystis_jiroveci	0.0	1.001	0.0	0.0	0.0	0.0	0.0	0.0	0.0	0.0	1.002	0.0	0.0	0.0	0.0	0.0	0.0	0.0
Pseudomonas_aeruginosa	0.0	1.001	0.0	0.0	0.0	0.0	0.0	0.0	0.0	0.0	1.002	0.0	0.0	0.0	0.0	0.0	0.0	0.0
Serratia_marcescens	0.143	1.001	14.2857142857	0.0	0.0	0.0	0.0	0.0	0.0	0.0	1.002	0.0	0.0	0.0	0.0	0.0	0.0	0.0
Staphylococcus_aureus	0.0	1.001	0.0	0.0	0.0	0.0	0.0	0.0	0.0	0.0	1.002	0.0	0.0	0.0	0.0	0.0	0.0	0.0
Stenotrophomonas_maltophilia	0.0	1.001	0.0	0.0	0.0	0.0	0.0	0.0	0.0	0.0	1.002	0.0	0.0	0.0	0.0	0.0	0.0	0.0
Streptococcus_pneumoniae	0.0	1.001	0.0	0.0	0.0	0.0	0.0	0.0	0.0	0.167	1.002	16.6666666667	0.0	0.0	0.0	0.0	0.0	0.0
TTVlikeminivirus	0.0	1.001	0.0	0.0	0.0	0.0	0.0	0.0	0.0	0.0	1.002	0.0	0.0	0.0	0.0	0.0	0.0	0.0

SampleID	Patient26	Patient26: parent seq. count	Patient26: rel. freq. (%)	Patient27	Patient27: parent seq. count	Patient27: rel. freq. (%)	Patient28	Patient28: parent seq. count	Patient28: rel. freq. (%)	Patient29	Patient29: parent seq. count	Patient29: rel. freq. (%)	Patient3	Patient3: parent seq. count	Patient3: rel. freq. (%)	Patient30	Patient30: parent seq. count	Patient30: rel. freq. (%)
Acinetobacter_baumannii	0.0	1.002	0.0	0.0	0.0	0.0	0.0	0.0	0.0	0.0	0.0	0.0	0.0	0.0	0.0	0.0	0.0	0.0
Acinetobacter_saimannii	0.0	1.002	0.0	0.0	0.0	0.0	0.0	0.0	0.0	0.0	0.0	0.0	0.0	0.0	0.0	0.0	0.0	0.0
Aspergillus_flavus	0.167	1.002	16.6666666667	0.0	0.0	0.0	0.0	0.0	0.0	0.0	0.0	0.0	0.0	0.0	0.0	0.0	0.0	0.0
Aspergillus_fumigatus	0.0	1.002	0.0	0.0	0.0	0.0	0.0	0.0	0.0	0.0	0.0	0.0	0.0	0.0	0.0	0.0	0.0	0.0
Aspergillus_niger	0.0	1.002	0.0	0.0	0.0	0.0	0.0	0.0	0.0	0.0	0.0	0.0	0.0	0.0	0.0	0.0	0.0	0.0
Candida_albicans	0.167	1.002	16.6666666667	0.0	0.0	0.0	0.0	0.0	0.0	0.0	0.0	0.0	0.0	0.0	0.0	0.0	0.0	0.0
Candida_tropicalis	0.0	1.002	0.0	0.0	0.0	0.0	0.0	0.0	0.0	0.0	0.0	0.0	0.0	0.0	0.0	0.0	0.0	0.0
CMV	0.0	1.002	0.0	0.0	0.0	0.0	0.0	0.0	0.0	0.0	0.0	0.0	0.0	0.0	0.0	0.0	0.0	0.0
Corynebacterium_striatum	0.0	1.002	0.0	0.0	0.0	0.0	0.0	0.0	0.0	0.0	0.0	0.0	0.0	0.0	0.0	0.0	0.0	0.0
enterococcus_faecium	0.0	1.002	0.0	0.0	0.0	0.0	0.0	0.0	0.0	0.0	0.0	0.0	0.0	0.0	0.0	0.0	0.0	0.0
Escherichia_coli	0.0	1.002	0.0	0.0	0.0	0.0	0.0	0.0	0.0	0.0	0.0	0.0	0.0	0.0	0.0	0.0	0.0	0.0
Haemophilus_haemolyticus	0.0	1.002	0.0	0.0	0.0	0.0	0.0	0.0	0.0	0.0	0.0	0.0	0.0	0.0	0.0	0.0	0.0	0.0
Haemophilus_influenzae	0.0	1.002	0.0	0.0	0.0	0.0	0.0	0.0	0.0	0.0	0.0	0.0	0.0	0.0	0.0	0.0	0.0	0.0
hepatitis_B_virus	0.167	1.002	16.6666666667	0.0	0.0	0.0	0.0	0.0	0.0	0.0	0.0	0.0	0.0	0.0	0.0	0.0	0.0	0.0
Hospital _Acinetobacter	0.0	1.002	0.0	0.0	0.0	0.0	0.0	0.0	0.0	0.0	0.0	0.0	0.0	0.0	0.0	0.0	0.0	0.0
human_beings_;Á_Herpesvirus1	0.0	1.002	0.0	0.0	0.0	0.0	0.0	0.0	0.0	0.0	0.0	0.0	0.0	0.0	0.0	0.0	0.0	0.0
human_beings_;Á_Herpesvirus5	0.167	1.002	16.6666666667	0.0	0.0	0.0	0.0	0.0	0.0	0.0	0.0	0.0	0.0	0.0	0.0	0.0	0.0	0.0
human_beings_;Á_Herpesvirus7	0.0	1.002	0.0	0.0	0.0	0.0	0.0	0.0	0.0	0.0	0.0	0.0	0.0	0.0	0.0	0.0	0.0	0.0
Human_herpesvirus4	0.0	1.002	0.0	0.0	0.0	0.0	0.0	0.0	0.0	0.0	0.0	0.0	0.0	0.0	0.0	0.0	0.0	0.0
Human_papillomavirus_type16	0.167	1.002	16.6666666667	0.0	0.0	0.0	0.0	0.0	0.0	0.0	0.0	0.0	0.0	0.0	0.0	0.0	0.0	0.0
klebsiella_pneumoniae	0.167	1.002	16.6666666667	0.0	0.0	0.0	0.0	0.0	0.0	0.0	0.0	0.0	0.0	0.0	0.0	0.0	0.0	0.0
M.mageritense	0.0	1.002	0.0	0.0	0.0	0.0	0.0	0.0	0.0	0.0	0.0	0.0	0.0	0.0	0.0	0.0	0.0	0.0
M.wolinskyi	0.0	1.002	0.0	0.0	0.0	0.0	0.0	0.0	0.0	0.0	0.0	0.0	0.0	0.0	0.0	0.0	0.0	0.0
Microcyclic_virus	0.0	1.002	0.0	0.0	0.0	0.0	0.0	0.0	0.0	0.0	0.0	0.0	0.0	0.0	0.0	0.0	0.0	0.0
Mycobacterium_Archaea	0.0	1.002	0.0	0.0	0.0	0.0	0.0	0.0	0.0	0.0	0.0	0.0	0.0	0.0	0.0	0.0	0.0	0.0
Mycobacterium_smegmatis	0.0	1.002	0.0	0.0	0.0	0.0	0.0	0.0	0.0	0.0	0.0	0.0	0.0	0.0	0.0	0.0	0.0	0.0
Neisseria_meningitidis	0.0	1.002	0.0	0.0	0.0	0.0	0.0	0.0	0.0	0.0	0.0	0.0	0.0	0.0	0.0	0.0	0.0	0.0
Pneumocystis_jiroveci	0.0	1.002	0.0	0.0	0.0	0.0	0.0	0.0	0.0	0.0	0.0	0.0	0.0	0.0	0.0	0.0	0.0	0.0
Pseudomonas_aeruginosa	0.0	1.002	0.0	0.0	0.0	0.0	0.0	0.0	0.0	0.0	0.0	0.0	0.0	0.0	0.0	0.0	0.0	0.0
Serratia_marcescens	0.0	1.002	0.0	0.0	0.0	0.0	0.0	0.0	0.0	0.0	0.0	0.0	0.0	0.0	0.0	0.0	0.0	0.0
Staphylococcus_aureus	0.0	1.002	0.0	0.0	0.0	0.0	0.0	0.0	0.0	0.0	0.0	0.0	0.0	0.0	0.0	0.0	0.0	0.0
Stenotrophomonas_maltophilia	0.0	1.002	0.0	0.0	0.0	0.0	0.0	0.0	0.0	0.0	0.0	0.0	0.0	0.0	0.0	0.0	0.0	0.0
Streptococcus_pneumoniae	0.0	1.002	0.0	0.0	0.0	0.0	0.0	0.0	0.0	0.0	0.0	0.0	0.0	0.0	0.0	0.0	0.0	0.0
TTVlikeminivirus	0.0	1.002	0.0	0.0	0.0	0.0	0.0	0.0	0.0	0.0	0.0	0.0	0.0	0.0	0.0	0.0	0.0	0.0

SampleID	Patient4	Patient4: parent seq. count	Patient4: rel. freq. (%)	Patient5	Patient5: parent seq. count	Patient5: rel. freq. (%)	Patient6	Patient6: parent seq. count	Patient6: rel. freq. (%)	Patient7	Patient7: parent seq. count	Patient7: rel. freq. (%)	Patient8	Patient8: parent seq. count	Patient8: rel. freq. (%)	Patient9	Patient9: parent seq. count	Patient9: rel. freq. (%)
Acinetobacter_baumannii	0.0	0.0	0.0	0.0	1.002	0.0	0.0	0.0	0.0	0.0	0.0	0.0	0.333	0.999	33.3333333333	0.0	0.999	0.0
Acinetobacter_saimannii	0.0	0.0	0.0	0.0	1.002	0.0	0.0	0.0	0.0	0.0	0.0	0.0	0.0	0.999	0.0	0.0	0.999	0.0
Aspergillus_flavus	0.0	0.0	0.0	0.0	1.002	0.0	0.0	0.0	0.0	0.0	0.0	0.0	0.0	0.999	0.0	0.0	0.999	0.0
Aspergillus_fumigatus	0.0	0.0	0.0	0.0	1.002	0.0	0.0	0.0	0.0	0.0	0.0	0.0	0.0	0.999	0.0	0.333	0.999	33.3333333333
Aspergillus_niger	0.0	0.0	0.0	0.167	1.002	16.6666666667	0.0	0.0	0.0	0.0	0.0	0.0	0.0	0.999	0.0	0.0	0.999	0.0
Candida_albicans	0.0	0.0	0.0	0.0	1.002	0.0	0.0	0.0	0.0	0.0	0.0	0.0	0.333	0.999	33.3333333333	0.0	0.999	0.0
Candida_tropicalis	0.0	0.0	0.0	0.0	1.002	0.0	0.0	0.0	0.0	0.0	0.0	0.0	0.0	0.999	0.0	0.0	0.999	0.0
CMV	0.0	0.0	0.0	0.0	1.002	0.0	0.0	0.0	0.0	0.0	0.0	0.0	0.0	0.999	0.0	0.0	0.999	0.0
Corynebacterium_striatum	0.0	0.0	0.0	0.0	1.002	0.0	0.0	0.0	0.0	0.0	0.0	0.0	0.0	0.999	0.0	0.333	0.999	33.3333333333
enterococcus_faecium	0.0	0.0	0.0	0.0	1.002	0.0	0.0	0.0	0.0	0.0	0.0	0.0	0.0	0.999	0.0	0.0	0.999	0.0
Escherichia_coli	0.0	0.0	0.0	0.0	1.002	0.0	0.0	0.0	0.0	0.0	0.0	0.0	0.0	0.999	0.0	0.0	0.999	0.0
Haemophilus_haemolyticus	0.0	0.0	0.0	0.167	1.002	16.6666666667	0.0	0.0	0.0	0.0	0.0	0.0	0.0	0.999	0.0	0.0	0.999	0.0
Haemophilus_influenzae	0.0	0.0	0.0	0.167	1.002	16.6666666667	0.0	0.0	0.0	0.0	0.0	0.0	0.0	0.999	0.0	0.0	0.999	0.0
hepatitis_B_virus	0.0	0.0	0.0	0.0	1.002	0.0	0.0	0.0	0.0	0.0	0.0	0.0	0.0	0.999	0.0	0.0	0.999	0.0
Hospital _Acinetobacter	0.0	0.0	0.0	0.0	1.002	0.0	0.0	0.0	0.0	0.0	0.0	0.0	0.0	0.999	0.0	0.0	0.999	0.0
human_beings_;Á_Herpesvirus1	0.0	0.0	0.0	0.0	1.002	0.0	0.0	0.0	0.0	0.0	0.0	0.0	0.0	0.999	0.0	0.0	0.999	0.0
human_beings_;Á_Herpesvirus5	0.0	0.0	0.0	0.0	1.002	0.0	0.0	0.0	0.0	0.0	0.0	0.0	0.0	0.999	0.0	0.0	0.999	0.0
human_beings_;Á_Herpesvirus7	0.0	0.0	0.0	0.0	1.002	0.0	0.0	0.0	0.0	0.0	0.0	0.0	0.0	0.999	0.0	0.0	0.999	0.0
Human_herpesvirus4	0.0	0.0	0.0	0.0	1.002	0.0	0.0	0.0	0.0	0.0	0.0	0.0	0.0	0.999	0.0	0.0	0.999	0.0
Human_papillomavirus_type16	0.0	0.0	0.0	0.0	1.002	0.0	0.0	0.0	0.0	0.0	0.0	0.0	0.0	0.999	0.0	0.0	0.999	0.0
klebsiella_pneumoniae	0.0	0.0	0.0	0.0	1.002	0.0	0.0	0.0	0.0	0.0	0.0	0.0	0.333	0.999	33.3333333333	0.0	0.999	0.0
M.mageritense	0.0	0.0	0.0	0.0	1.002	0.0	0.0	0.0	0.0	0.0	0.0	0.0	0.0	0.999	0.0	0.0	0.999	0.0
M.wolinskyi	0.0	0.0	0.0	0.0	1.002	0.0	0.0	0.0	0.0	0.0	0.0	0.0	0.0	0.999	0.0	0.0	0.999	0.0
Microcyclic_virus	0.0	0.0	0.0	0.0	1.002	0.0	0.0	0.0	0.0	0.0	0.0	0.0	0.0	0.999	0.0	0.333	0.999	33.3333333333
Mycobacterium_Archaea	0.0	0.0	0.0	0.0	1.002	0.0	0.0	0.0	0.0	0.0	0.0	0.0	0.0	0.999	0.0	0.0	0.999	0.0
Mycobacterium_smegmatis	0.0	0.0	0.0	0.0	1.002	0.0	0.0	0.0	0.0	0.0	0.0	0.0	0.0	0.999	0.0	0.0	0.999	0.0
Neisseria_meningitidis	0.0	0.0	0.0	0.167	1.002	16.6666666667	0.0	0.0	0.0	0.0	0.0	0.0	0.0	0.999	0.0	0.0	0.999	0.0
Pneumocystis_jiroveci	0.0	0.0	0.0	0.167	1.002	16.6666666667	0.0	0.0	0.0	0.0	0.0	0.0	0.0	0.999	0.0	0.0	0.999	0.0
Pseudomonas_aeruginosa	0.0	0.0	0.0	0.167	1.002	16.6666666667	0.0	0.0	0.0	0.0	0.0	0.0	0.0	0.999	0.0	0.0	0.999	0.0
Serratia_marcescens	0.0	0.0	0.0	0.0	1.002	0.0	0.0	0.0	0.0	0.0	0.0	0.0	0.0	0.999	0.0	0.0	0.999	0.0
Staphylococcus_aureus	0.0	0.0	0.0	0.0	1.002	0.0	0.0	0.0	0.0	0.0	0.0	0.0	0.0	0.999	0.0	0.0	0.999	0.0
Stenotrophomonas_maltophilia	0.0	0.0	0.0	0.0	1.002	0.0	0.0	0.0	0.0	0.0	0.0	0.0	0.0	0.999	0.0	0.0	0.999	0.0
Streptococcus_pneumoniae	0.0	0.0	0.0	0.0	1.002	0.0	0.0	0.0	0.0	0.0	0.0	0.0	0.0	0.999	0.0	0.0	0.999	0.0
TTVlikeminivirus	0.0	0.0	0.0	0.0	1.002	0.0	0.0	0.0	0.0	0.0	0.0	0.0	0.0	0.999	0.0	0.0	0.999	0.0

SampleID	Patient31	Patient31: parent seq. count	Patient31: rel. freq. (%)	Patient32	Patient32: parent seq. count	Patient32: rel. freq. (%)	Patient33	Patient33: parent seq. count	Patient33: rel. freq. (%)	Patient34	Patient34: parent seq. count	Patient34: rel. freq. (%)	Patient35	Patient35: parent seq. count	Patient35: rel. freq. (%)	Patient36	Patient36: parent seq. count	Patient36: rel. freq. (%)
Acinetobacter_baumannii	0.0	0.0	0.0	0.0	0.0	0.0	0.33	0.99	33.3333333333	0.143	1.001	14.2857142857	0.0	0.0	0.0	0.0	0.0	0.0
Acinetobacter_saimannii	0.0	0.0	0.0	0.0	0.0	0.0	0.0	0.99	0.0	0.143	1.001	14.2857142857	0.0	0.0	0.0	0.0	0.0	0.0
Aspergillus_flavus	0.0	0.0	0.0	0.0	0.0	0.0	0.0	0.99	0.0	0.0	1.001	0.0	0.0	0.0	0.0	0.0	0.0	0.0
Aspergillus_fumigatus	0.0	0.0	0.0	0.0	0.0	0.0	0.0	0.99	0.0	0.0	1.001	0.0	0.0	0.0	0.0	0.0	0.0	0.0
Aspergillus_niger	0.0	0.0	0.0	0.0	0.0	0.0	0.0	0.99	0.0	0.0	1.001	0.0	0.0	0.0	0.0	0.0	0.0	0.0
Candida_albicans	0.0	0.0	0.0	0.0	0.0	0.0	0.0	0.99	0.0	0.143	1.001	14.2857142857	0.0	0.0	0.0	0.0	0.0	0.0
Candida_tropicalis	0.0	0.0	0.0	0.0	0.0	0.0	0.0	0.99	0.0	0.0	1.001	0.0	0.0	0.0	0.0	0.0	0.0	0.0
CMV	0.0	0.0	0.0	0.0	0.0	0.0	0.0	0.99	0.0	0.0	1.001	0.0	0.0	0.0	0.0	0.0	0.0	0.0
Corynebacterium_striatum	0.0	0.0	0.0	0.0	0.0	0.0	0.0	0.99	0.0	0.0	1.001	0.0	0.0	0.0	0.0	0.0	0.0	0.0
enterococcus_faecium	0.0	0.0	0.0	0.0	0.0	0.0	0.0	0.99	0.0	0.0	1.001	0.0	0.0	0.0	0.0	0.0	0.0	0.0
Escherichia_coli	0.0	0.0	0.0	0.0	0.0	0.0	0.0	0.99	0.0	0.143	1.001	14.2857142857	0.0	0.0	0.0	0.0	0.0	0.0
Haemophilus_haemolyticus	0.0	0.0	0.0	0.0	0.0	0.0	0.0	0.99	0.0	0.0	1.001	0.0	0.0	0.0	0.0	0.0	0.0	0.0
Haemophilus_influenzae	0.0	0.0	0.0	0.0	0.0	0.0	0.0	0.99	0.0	0.0	1.001	0.0	0.0	0.0	0.0	0.0	0.0	0.0
hepatitis_B_virus	0.0	0.0	0.0	0.0	0.0	0.0	0.0	0.99	0.0	0.0	1.001	0.0	0.0	0.0	0.0	0.0	0.0	0.0
Hospital _Acinetobacter	0.0	0.0	0.0	0.0	0.0	0.0	0.0	0.99	0.0	0.143	1.001	14.2857142857	0.0	0.0	0.0	0.0	0.0	0.0
human_beings_;Á_Herpesvirus1	0.0	0.0	0.0	0.0	0.0	0.0	0.0	0.99	0.0	0.0	1.001	0.0	0.0	0.0	0.0	0.0	0.0	0.0
human_beings_;Á_Herpesvirus5	0.0	0.0	0.0	0.0	0.0	0.0	0.33	0.99	33.3333333333	0.0	1.001	0.0	0.0	0.0	0.0	0.0	0.0	0.0
human_beings_;Á_Herpesvirus7	0.0	0.0	0.0	0.0	0.0	0.0	0.0	0.99	0.0	0.0	1.001	0.0	0.0	0.0	0.0	0.0	0.0	0.0
Human_herpesvirus4	0.0	0.0	0.0	0.0	0.0	0.0	0.0	0.99	0.0	0.0	1.001	0.0	0.0	0.0	0.0	0.0	0.0	0.0
Human_papillomavirus_type16	0.0	0.0	0.0	0.0	0.0	0.0	0.0	0.99	0.0	0.0	1.001	0.0	0.0	0.0	0.0	0.0	0.0	0.0
klebsiella_pneumoniae	0.0	0.0	0.0	0.0	0.0	0.0	0.0	0.99	0.0	0.143	1.001	14.2857142857	0.0	0.0	0.0	0.0	0.0	0.0
M.mageritense	0.0	0.0	0.0	0.0	0.0	0.0	0.0	0.99	0.0	0.0	1.001	0.0	0.0	0.0	0.0	0.0	0.0	0.0
M.wolinskyi	0.0	0.0	0.0	0.0	0.0	0.0	0.0	0.99	0.0	0.0	1.001	0.0	0.0	0.0	0.0	0.0	0.0	0.0
Microcyclic_virus	0.0	0.0	0.0	0.0	0.0	0.0	0.0	0.99	0.0	0.0	1.001	0.0	0.0	0.0	0.0	0.0	0.0	0.0
Mycobacterium_Archaea	0.0	0.0	0.0	0.0	0.0	0.0	0.0	0.99	0.0	0.0	1.001	0.0	0.0	0.0	0.0	0.0	0.0	0.0
Mycobacterium_smegmatis	0.0	0.0	0.0	0.0	0.0	0.0	0.0	0.99	0.0	0.0	1.001	0.0	0.0	0.0	0.0	0.0	0.0	0.0
Neisseria_meningitidis	0.0	0.0	0.0	0.0	0.0	0.0	0.0	0.99	0.0	0.0	1.001	0.0	0.0	0.0	0.0	0.0	0.0	0.0
Pneumocystis_jiroveci	0.0	0.0	0.0	0.0	0.0	0.0	0.33	0.99	33.3333333333	0.0	1.001	0.0	0.0	0.0	0.0	0.0	0.0	0.0
Pseudomonas_aeruginosa	0.0	0.0	0.0	0.0	0.0	0.0	0.0	0.99	0.0	0.0	1.001	0.0	0.0	0.0	0.0	0.0	0.0	0.0
Serratia_marcescens	0.0	0.0	0.0	0.0	0.0	0.0	0.0	0.99	0.0	0.143	1.001	14.2857142857	0.0	0.0	0.0	0.0	0.0	0.0
Staphylococcus_aureus	0.0	0.0	0.0	0.0	0.0	0.0	0.0	0.99	0.0	0.0	1.001	0.0	0.0	0.0	0.0	0.0	0.0	0.0
Stenotrophomonas_maltophilia	0.0	0.0	0.0	0.0	0.0	0.0	0.0	0.99	0.0	0.0	1.001	0.0	0.0	0.0	0.0	0.0	0.0	0.0
Streptococcus_pneumoniae	0.0	0.0	0.0	0.0	0.0	0.0	0.0	0.99	0.0	0.0	1.001	0.0	0.0	0.0	0.0	0.0	0.0	0.0
TTVlikeminivirus	0.0	0.0	0.0	0.0	0.0	0.0	0.0	0.99	0.0	0.0	1.001	0.0	0.0	0.0	0.0	0.0	0.0	0.0

SampleID	Patient37	Patient37: parent seq. count	Patient37: rel. freq. (%)	Patient38	Patient38: parent seq. count	Patient38: rel. freq. (%)	Patient39	Patient39: parent seq. count	Patient39: rel. freq. (%)	Patient40	Patient40: parent seq. count	Patient40: rel. freq. (%)	Patient41	Patient41: parent seq. count	Patient41: rel. freq. (%)	Patient42	Patient42: parent seq. count	Patient42: rel. freq. (%)
Acinetobacter_baumannii	0.0	0.0	0.0	0.0	0.0	0.0	0.0	1.25	0.0	0.0	1.0	0.0	0.0	0.0	0.0	0.0	0.0	0.0
Acinetobacter_saimannii	0.0	0.0	0.0	0.0	0.0	0.0	0.0	1.25	0.0	0.0	1.0	0.0	0.0	0.0	0.0	0.0	0.0	0.0
Aspergillus_flavus	0.0	0.0	0.0	0.0	0.0	0.0	0.0	1.25	0.0	0.5	1.0	50.0	0.0	0.0	0.0	0.0	0.0	0.0
Aspergillus_fumigatus	0.0	0.0	0.0	0.0	0.0	0.0	0.0	1.25	0.0	0.0	1.0	0.0	0.0	0.0	0.0	0.0	0.0	0.0
Aspergillus_niger	0.0	0.0	0.0	0.0	0.0	0.0	0.0	1.25	0.0	0.0	1.0	0.0	0.0	0.0	0.0	0.0	0.0	0.0
Candida_albicans	0.0	0.0	0.0	0.0	0.0	0.0	0.0	1.25	0.0	0.0	1.0	0.0	0.0	0.0	0.0	0.0	0.0	0.0
Candida_tropicalis	0.0	0.0	0.0	0.0	0.0	0.0	0.0	1.25	0.0	0.0	1.0	0.0	0.0	0.0	0.0	0.0	0.0	0.0
CMV	0.0	0.0	0.0	0.0	0.0	0.0	0.25	1.25	20.0	0.0	1.0	0.0	0.0	0.0	0.0	0.0	0.0	0.0
Corynebacterium_striatum	0.0	0.0	0.0	0.0	0.0	0.0	0.0	1.25	0.0	0.0	1.0	0.0	0.0	0.0	0.0	0.0	0.0	0.0
enterococcus_faecium	0.0	0.0	0.0	0.0	0.0	0.0	0.0	1.25	0.0	0.0	1.0	0.0	0.0	0.0	0.0	0.0	0.0	0.0
Escherichia_coli	0.0	0.0	0.0	0.0	0.0	0.0	0.0	1.25	0.0	0.0	1.0	0.0	0.0	0.0	0.0	0.0	0.0	0.0
Haemophilus_haemolyticus	0.0	0.0	0.0	0.0	0.0	0.0	0.0	1.25	0.0	0.0	1.0	0.0	0.0	0.0	0.0	0.0	0.0	0.0
Haemophilus_influenzae	0.0	0.0	0.0	0.0	0.0	0.0	0.0	1.25	0.0	0.0	1.0	0.0	0.0	0.0	0.0	0.0	0.0	0.0
hepatitis_B_virus	0.0	0.0	0.0	0.0	0.0	0.0	0.0	1.25	0.0	0.0	1.0	0.0	0.0	0.0	0.0	0.0	0.0	0.0
Hospital _Acinetobacter	0.0	0.0	0.0	0.0	0.0	0.0	0.0	1.25	0.0	0.0	1.0	0.0	0.0	0.0	0.0	0.0	0.0	0.0
human_beings_;Á_Herpesvirus1	0.0	0.0	0.0	0.0	0.0	0.0	0.25	1.25	20.0	0.0	1.0	0.0	0.0	0.0	0.0	0.0	0.0	0.0
human_beings_;Á_Herpesvirus5	0.0	0.0	0.0	0.0	0.0	0.0	0.0	1.25	0.0	0.0	1.0	0.0	0.0	0.0	0.0	0.0	0.0	0.0
human_beings_;Á_Herpesvirus7	0.0	0.0	0.0	0.0	0.0	0.0	0.25	1.25	20.0	0.0	1.0	0.0	0.0	0.0	0.0	0.0	0.0	0.0
Human_herpesvirus4	0.0	0.0	0.0	0.0	0.0	0.0	0.0	1.25	0.0	0.0	1.0	0.0	0.0	0.0	0.0	0.0	0.0	0.0
Human_papillomavirus_type16	0.0	0.0	0.0	0.0	0.0	0.0	0.0	1.25	0.0	0.0	1.0	0.0	0.0	0.0	0.0	0.0	0.0	0.0
klebsiella_pneumoniae	0.0	0.0	0.0	0.0	0.0	0.0	0.25	1.25	20.0	0.0	1.0	0.0	0.0	0.0	0.0	0.0	0.0	0.0
M.mageritense	0.0	0.0	0.0	0.0	0.0	0.0	0.0	1.25	0.0	0.0	1.0	0.0	0.0	0.0	0.0	0.0	0.0	0.0
M.wolinskyi	0.0	0.0	0.0	0.0	0.0	0.0	0.0	1.25	0.0	0.0	1.0	0.0	0.0	0.0	0.0	0.0	0.0	0.0
Microcyclic_virus	0.0	0.0	0.0	0.0	0.0	0.0	0.0	1.25	0.0	0.5	1.0	50.0	0.0	0.0	0.0	0.0	0.0	0.0
Mycobacterium_Archaea	0.0	0.0	0.0	0.0	0.0	0.0	0.0	1.25	0.0	0.0	1.0	0.0	0.0	0.0	0.0	0.0	0.0	0.0
Mycobacterium_smegmatis	0.0	0.0	0.0	0.0	0.0	0.0	0.0	1.25	0.0	0.0	1.0	0.0	0.0	0.0	0.0	0.0	0.0	0.0
Neisseria_meningitidis	0.0	0.0	0.0	0.0	0.0	0.0	0.0	1.25	0.0	0.0	1.0	0.0	0.0	0.0	0.0	0.0	0.0	0.0
Pneumocystis_jiroveci	0.0	0.0	0.0	0.0	0.0	0.0	0.0	1.25	0.0	0.0	1.0	0.0	0.0	0.0	0.0	0.0	0.0	0.0
Pseudomonas_aeruginosa	0.0	0.0	0.0	0.0	0.0	0.0	0.0	1.25	0.0	0.0	1.0	0.0	0.0	0.0	0.0	0.0	0.0	0.0
Serratia_marcescens	0.0	0.0	0.0	0.0	0.0	0.0	0.0	1.25	0.0	0.0	1.0	0.0	0.0	0.0	0.0	0.0	0.0	0.0
Staphylococcus_aureus	0.0	0.0	0.0	0.0	0.0	0.0	0.0	1.25	0.0	0.0	1.0	0.0	0.0	0.0	0.0	0.0	0.0	0.0
Stenotrophomonas_maltophilia	0.0	0.0	0.0	0.0	0.0	0.0	0.25	1.25	20.0	0.0	1.0	0.0	0.0	0.0	0.0	0.0	0.0	0.0
Streptococcus_pneumoniae	0.0	0.0	0.0	0.0	0.0	0.0	0.0	1.25	0.0	0.0	1.0	0.0	0.0	0.0	0.0	0.0	0.0	0.0
TTVlikeminivirus	0.0	0.0	0.0	0.0	0.0	0.0	0.0	1.25	0.0	0.0	1.0	0.0	0.0	0.0	0.0	0.0	0.0	0.0

SampleID	Patient43	Patient43: parent seq. count	Patient43: rel. freq. (%)	Patient44	Patient44: parent seq. count	Patient44: rel. freq. (%)	Patient45	Patient45: parent seq. count	Patient45: rel. freq. (%)	Patient46	Patient46: parent seq. count	Patient46: rel. freq. (%)
Acinetobacter_baumannii	0.0	0.0	0.0	0.0	1.0	0.0	0.0	0.0	0.0	0.0	0.0	0.0
Acinetobacter_saimannii	0.0	0.0	0.0	0.0	1.0	0.0	0.0	0.0	0.0	0.0	0.0	0.0
Aspergillus_flavus	0.0	0.0	0.0	0.0	1.0	0.0	0.0	0.0	0.0	0.0	0.0	0.0
Aspergillus_fumigatus	0.0	0.0	0.0	0.0	1.0	0.0	0.0	0.0	0.0	0.0	0.0	0.0
Aspergillus_niger	0.0	0.0	0.0	0.0	1.0	0.0	0.0	0.0	0.0	0.0	0.0	0.0
Candida_albicans	0.0	0.0	0.0	0.0	1.0	0.0	0.0	0.0	0.0	0.0	0.0	0.0
Candida_tropicalis	0.0	0.0	0.0	0.25	1.0	25.0	0.0	0.0	0.0	0.0	0.0	0.0
CMV	0.0	0.0	0.0	0.0	1.0	0.0	0.0	0.0	0.0	0.0	0.0	0.0
Corynebacterium_striatum	0.0	0.0	0.0	0.0	1.0	0.0	0.0	0.0	0.0	0.0	0.0	0.0
enterococcus_faecium	0.0	0.0	0.0	0.0	1.0	0.0	0.0	0.0	0.0	0.0	0.0	0.0
Escherichia_coli	0.0	0.0	0.0	0.0	1.0	0.0	0.0	0.0	0.0	0.0	0.0	0.0
Haemophilus_haemolyticus	0.0	0.0	0.0	0.0	1.0	0.0	0.0	0.0	0.0	0.0	0.0	0.0
Haemophilus_influenzae	0.0	0.0	0.0	0.0	1.0	0.0	0.0	0.0	0.0	0.0	0.0	0.0
hepatitis_B_virus	0.0	0.0	0.0	0.0	1.0	0.0	0.0	0.0	0.0	0.0	0.0	0.0
Hospital _Acinetobacter	0.0	0.0	0.0	0.0	1.0	0.0	0.0	0.0	0.0	0.0	0.0	0.0
human_beings_;Á_Herpesvirus1	0.0	0.0	0.0	0.0	1.0	0.0	0.0	0.0	0.0	0.0	0.0	0.0
human_beings_;Á_Herpesvirus5	0.0	0.0	0.0	0.25	1.0	25.0	0.0	0.0	0.0	0.0	0.0	0.0
human_beings_;Á_Herpesvirus7	0.0	0.0	0.0	0.0	1.0	0.0	0.0	0.0	0.0	0.0	0.0	0.0
Human_herpesvirus4	0.0	0.0	0.0	0.0	1.0	0.0	0.0	0.0	0.0	0.0	0.0	0.0
Human_papillomavirus_type16	0.0	0.0	0.0	0.0	1.0	0.0	0.0	0.0	0.0	0.0	0.0	0.0
klebsiella_pneumoniae	0.0	0.0	0.0	0.0	1.0	0.0	0.0	0.0	0.0	0.0	0.0	0.0
M.mageritense	0.0	0.0	0.0	0.0	1.0	0.0	0.0	0.0	0.0	0.0	0.0	0.0
M.wolinskyi	0.0	0.0	0.0	0.0	1.0	0.0	0.0	0.0	0.0	0.0	0.0	0.0
Microcyclic_virus	0.0	0.0	0.0	0.25	1.0	25.0	0.0	0.0	0.0	0.0	0.0	0.0
Mycobacterium_Archaea	0.0	0.0	0.0	0.0	1.0	0.0	0.0	0.0	0.0	0.0	0.0	0.0
Mycobacterium_smegmatis	0.0	0.0	0.0	0.0	1.0	0.0	0.0	0.0	0.0	0.0	0.0	0.0
Neisseria_meningitidis	0.0	0.0	0.0	0.0	1.0	0.0	0.0	0.0	0.0	0.0	0.0	0.0
Pneumocystis_jiroveci	0.0	0.0	0.0	0.0	1.0	0.0	0.0	0.0	0.0	0.0	0.0	0.0
Pseudomonas_aeruginosa	0.0	0.0	0.0	0.0	1.0	0.0	0.0	0.0	0.0	0.0	0.0	0.0
Serratia_marcescens	0.0	0.0	0.0	0.0	1.0	0.0	0.0	0.0	0.0	0.0	0.0	0.0
Staphylococcus_aureus	0.0	0.0	0.0	0.0	1.0	0.0	0.0	0.0	0.0	0.0	0.0	0.0
Stenotrophomonas_maltophilia	0.0	0.0	0.0	0.0	1.0	0.0	0.0	0.0	0.0	0.0	0.0	0.0
Streptococcus_pneumoniae	0.0	0.0	0.0	0.0	1.0	0.0	0.0	0.0	0.0	0.0	0.0	0.0
TTVlikeminivirus	0.0	0.0	0.0	0.25	1.0	25.0	0.0	0.0	0.0	0.0	0.0	0.0
